# IFI16 promotes the progression of clear cell renal cell carcinoma through the IL6/PI3K/AKT axis

**DOI:** 10.1186/s12967-024-05354-w

**Published:** 2024-06-03

**Authors:** Ke Lu, Yan Zhao, Yu Li, Zhenyu Fu, Yongchang Chen, Ying Kong, Gang Li

**Affiliations:** 1 Department of Urology, The First Affiliated Hospital of Soochow University, Suzhou, 215000 Jiangsu China; 2 Department of Urology, Affiliated Changshu Hospital of Nantong University, Changshu, 215500 Jiangsu China; 3 Department of Urology, Xuzhou Cancer Hospital, Affiliated Hospital of Jiangsu University, Xuzhou, 221000 Jiangsu China

**Keywords:** IFI16, Clear cell renal cell carcinoma, EMT, PI3K/AKT, Tumor progression

## Abstract

**Background:**

Clear cell renal cell carcinoma (ccRCC) is a common disease in the urinary system, with a high incidence and poor prognosis in advanced stages. Although γ-interferon-inducible protein 16 (IFI16) has been reported to play a role in various tumors, its involvement in ccRCC remains poorly documented, and the molecular mechanisms are not yet clear.

**Methods:**

We conducted bioinformatics analysis to study the expression of IFI16 in ccRCC using public databases. Additionally, we analyzed and validated clinical specimens that we collected. Subsequently, we explored the impact of IFI16 on ccRCC cell proliferation, migration, and invasion through in vitro and in vivo experiments. Furthermore, we predicted downstream molecules and pathways using transcriptome analysis and confirmed them through follow-up experimental validation.

**Results:**

IFI16 was significantly upregulated in ccRCC tissue and correlated with poor patient prognosis. In vitro, IFI16 promoted ccRCC cell proliferation, migration, and invasion, while in vivo, it facilitated subcutaneous tumor growth and the formation of lung metastatic foci. Knocking down IFI16 suppressed its oncogenic function. At the molecular level, IFI16 promoted the transcription and translation of IL6, subsequently activating the PI3K/AKT signaling pathway and inducing epithelial-mesenchymal transition (EMT).

**Conclusion:**

IFI16 induced EMT through the IL6/PI3K/AKT axis, promoting the progression of ccRCC.

**Supplementary Information:**

The online version contains supplementary material available at 10.1186/s12967-024-05354-w.

## Background

Renal cell carcinoma (RCC) is one of the common tumors in the urinary system. Clear cell renal cell carcinoma (ccRCC) originates from the proximal renal tubules, accounting for approximately 85% of all kidney cancers [1]. For patients with advanced RCC, the overall prognosis remains poor [2]. Although most RCC patients were initially diagnosed with localized small tumors, some cases present as locally advanced, and 17% of patients were diagnosed with distant metastasis at their first visit [3]. Despite advances in imaging techniques and the clinical application of targeted drugs, there is still a need to explore new molecular mechanisms underlying RCC progression as potential novel targets for clinical applications.

γ-interferon-inducible protein 16 (IFI16) is a critical DNA sensor that triggers downstream STING-dependent type I interferon (IFN-I) production and has antiviral immune functions [4, 5]. Additionally, DNA-binding protein IFI16 can activate STING, leading to NF-κB signal transduction activation [6]. Recent research suggests that IFI16 also plays an important role in cancer. Kondo et al. discovered that IFI16 contributed to the progression of p53-inactivated oral squamous cell carcinoma [7]. Cai et al. reported that IFI16 upregulated PD-L1 in the immune microenvironment via the STING-TBK1-NF-κB pathway to promote cervical cancer progression [8]. In glioblastoma, Gao et al. found that IFI16 enhanced mesenchymal phenotype maintenance and increased radiotherapy resistance by activating NF-κB and STAT3 signaling pathways [9]. Ga et al. confirmed that IFI16 induced macrophages to secrete inflammatory cytokines and promoted breast cancer tumor growth [10]. However, there were also reports that IFI16 inhibited tumor growth in hepatocellular carcinoma by activating p53 signaling and inflammasomes [11]. A study by Alimirah et al. revealed that increased IFI16 expression in prostate cancer cells was associated with reduced androgen receptor expression and cell apoptosis [12]. These findings indicate that IFI16 may have different roles in different tumors. However, there is limited information on the biological function of IFI16 in RCC, and its underlying mechanisms remain unexplored. Therefore, we aim to investigate whether IFI16 influences RCC progression and explore its mechanisms.

Interleukin-6 (IL6) is a glycosylated polypeptide chain and a crucial cytokine involved in various aspects of inflammation and immune regulation [13–16]. In the tumor microenvironment, IL6 serves as a major cell factor and participates in the development of several cancers. It is highly expressed and has pro-cancer effects in colorectal cancer, breast cancer, lung cancer, and liver cancer [17–21]. IL6 binds to the IL6 receptor (IL6R) on cell membranes, activating a series of cell signaling pathways such as Ras/Raf/MEK/MAPK, PI3K/AKT, and JAK/STAT, leading to various cellular events [21–23]. The PI3K/AKT pathway, which is moderately mutated but highly activated in RCC, plays a crucial role in metabolic reprogramming and is a key target for RCC therapy [24, 25].

In our study, we observed differences in IFI16 expression related to clinical pathological features and survival rates in ccRCC patients. We demonstrated that IFI16 promotes ccRCC cell proliferation, migration, and invasion both in vitro and in vivo. Furthermore, we found that IFI16 upregulates IL6 expression, subsequently inducing PI3K/AKT pathway activation and epithelial-mesenchymal transition (EMT). Therefore, we propose that targeting IFI16 could be a potential therapeutic strategy for ccRCC patients.

## Materials and methods

### Bioinformatics analysis

The clinical characteristics and transcriptome data of KIRC patients were obtained from The Cancer Genome Atlas (TCGA, https://portal.gdc.cancer.gov/) database (542 tumor samples and 72 adjacent normal samples) and Gene Expression Omnibus (GEO, https://www.ncbi.nlm.nih.gov/geo/) database (GSE66272, GSE76351, GSE68417, GSE40435, GSE71963) [26–30]. The data of 565 KIRC samples were retained for further analysis after excluding those without survival information. R software (version 4.2.2, https://www.r-project.org/) was used for data analysis. The “limma” package was applied for the identification of differentially expressed genes (DEGs) between tumor and normal samples in TCGA database while the DEGs between tumor and normal samples in GEO database were got by GEO2R online. The DEGs were identified with a filter as log2|Fold change|> 1 and adjusted p < 0.05. The “ggplot2” and “ggpubr” package were used for visualization. The “survival” and “survminer” packages were used for conducting survival analysis on TCGA data.

### Patient specimens and clinical information

This study included 92 patients who underwent laparoscopic radical nephrectomy or laparoscopic partial nephrectomy at The First Affiliated Hospital of Soochow University from January 2017 to December 2022, and postoperative pathology confirmed renal clear cell carcinoma. The study was approved by the hospital ethics committee (2023-KY-SKJQ-01). Patients had not received any targeted drugs or immunotherapies before surgery, and written informed consent was obtained from all patients before participating in the study. Tissue specimens obtained from patients were immediately fixed in formalin, embedded in paraffin for immunohistochemistry (IHC) studies, and another portion was promptly stored in liquid nitrogen for subsequent RT-qPCR and western blotting (WB). Clinical pathology refers to the Fuhrman grading system and the 2017 American Joint Committee on Cancer (AJCC) Tumor-Node-Metastasis (TNM) staging for renal cell carcinoma [31, 32].

### Cell culture, treatment and construction of stable transgenic strains

Human RCC cell lines (769-P, 786-O, ACHN, Caki-1, OS-RC-2) and the human proximal tubular epithelial cell line HK-2 were acquired from the American Type Culture Collection (ATCC). These cell lines were cultured at 37 °C in a 5% CO_2_ atmosphere using the recommended culture media, which included 10% fetal bovine serum and 100 U/mL penicillin/streptomycin. 769-P, 786-O and OS-RC-2 cells were cultured in RPMI-1640 medium (Gibco, USA). ACHN and HK-2 cells were cultured in MEM medium (Gibco, USA) while the Caki-1 cells were cultured in McCoy’s 5A medium (Gibco, USA). By performing PCR amplification of the human IFI16 gene, it was cloned into the EF-1aF-puro shuttle vector. Co-transfection with packaging plasmids (pGag/Pol, pRev, pVSV-G, obtained from genepharma company, China) was carried out in HEK-293T cells to generate lentiviruses. After 48 h, the supernatant was collected to obtain lentiviruses overexpressing IFI16. Similarly, lentiviruses overexpressing IL6 were also obtained. The short hairpin RNA (shRNA) targeting IFI16 was cloned into the pLKO.1-neo vector, and co-transfection with packaging plasmids (pMD2.G and psPAX2) was performed in HEK-293T cells to generate lentiviruses with knockdown expression of IFI16. Likewise, lentiviruses with knockdown expression of IL6 were also obtained. Following lentiviral infection of cells, overexpressing strains were selected using puromycin, while knockdown strains were selected using neomycin. The shRNA sequences can be found in the supplementary materials.

### RNA isolation and RT-qPCR

Total RNA was extracted from tissues and cells using TRIzol (Invitrogen, USA), followed by reverse transcription into cDNA using the Hifair II 1st Strand cDNA Synthesis Kit (YEASEN, China). Subsequently, a reverse transcription quantitative polymerase chain reaction (RT-qPCR) reaction was performed using the Hieff qPCR SYBR Green master mix (YEASEN, China).

The cDNA required approximately 10 ng for RT-qPCR and the thermocycling conditions for qPCR were as follows: (1) initial denaturation: 95 °C for 5 min, (2) 40 cycles of denaturation: 95 °C for 10 s, annealing: 55 °C for 20 s, and extension: 72 °C for 30 s. All primers were purchased from Genewiz (China). GAPDH was chosen as the internal control for normalization and target gene expression levels were calculated using the 2−ΔΔCt method. The specific primer sequences for the reaction are provided in the supplementary materials.

### Immunohistochemistry (IHC) staining

The paraffin-embedded specimens were sectioned into 4 µm thick slices for immunohistochemical staining. Subsequently, these sections underwent manual dewaxing using xylene and were subjected to a series of washes in gradient alcohol (100%, 95%, 80%) and ddH_2_O. Following this, the sections underwent antigen retrieval in a sodium citrate solution under high temperature and high pressure, followed by additional washes in ddH_2_O and PBS. The samples were treated with 3% H_2_O_2_ at room temperature for 15 min and then washed again in PBS. Subsequently, 3% BSA was introduced, and the samples were incubated at 37 °C for 30 min. The designated antibody (67790-1-Ig, Proteintech, China) was added, and the samples were incubated overnight at 4 °C in the absence of light. Immunohistochemistry was conducted using the SP Rabbit & Mouse HRP Kit (DAB) (CWBIO, China).

### Western blotting

Cell lysis was performed using an enhanced RIPA lysis buffer (Beyotime, China) supplemented with protease inhibitors. Protein concentration was determined using the BCA assay kit (Beyotime, China). The proteins, separated through 10% sodium dodecyl sulfate polyacrylamide gel electrophoresis, were electro-transferred onto a polyvinylidene fluoride membrane. Continuing, block the membrane with 5% BSA for 1 h and incubate with a diluted primary antibody against target gene at 4 °C. Specific primary antibodies against IFI16 (67790-1-Ig, Proteintech), IL6 (21865-1-AP, Proteintech), GAPDH (60004-1-Ig, Proteintech), E-cadherin (20874-1-AP, Proteintech), N-cadherin (22018-1-AP, Proteintech), Vimentin (10366-1-AP, Proteintech), AKT (10176-2-AP, Proteintech), p-AKT (28731-1-AP, Proteintech), PI3K (A4992, ABclonal), p-PI3K (AP0427, ABclonal) were used. On the next day, incubate with an HRP-conjugated goat anti-mouse secondary antibody (FDM007, Fdbio science) or goat anti-rabbit (FDR007, Fdbio science) at room temperature for 1 h. Finally, visualize the immunocomplexes on the membrane using ECL reagent (Fdbio science, China) and quantify the band intensity using Image J software (version 1.51). All experiments were repeated at least three times.

### CCK-8 assay

In a 96-well plate, cells from the experimental and control groups, each containing approximately 2000 cells, were seeded. After cell adhesion, 10 μL of Cell Counting Kit-8 reagent (NCM Biotech, China) was added to each well. Subsequently, the plate was incubated in a cell culture incubator for 60–75 min, and the absorbance at 450 nm was measured using a microplate reader.

### EdU assay

The EdU detection kit was purchased from Beyotime company (C0078S, China). Approximately 2 × 10^5^ cells were seeded in a 12-well plate, and after overnight incubation, 10 μM EdU was added. The cells were then further incubated for 2–3 h in the incubator. Subsequently, the cells were fixed with 4% paraformaldehyde at room temperature for 15 min, followed by a 15-min incubation with 0.3% Triton X-100. After washing with PBS, the cells were incubated with 200 μL of Click reaction solution at room temperature for 30 min. Finally, the cells were incubated with 1 mL Hoechst 33,342 for 10 min. Images were captured using a Nikon TI2-d-PD inverted microscope (Japan).

### Scratch assay

After seeding cells into a 6-well plate and allowing them to adhere, cell monolayers were scratched using a 10 μL pipette tip. Following the scratch, cells were washed with PBS and then cultured in medium containing 1% fetal bovine serum. Scratch assays were observed at 0 and 24 h, and cell migration was observed using an inverted microscope. We employed ImageJ software to calculate the area of the scratch at both 0 h and 24 h after scratching. The migration rate of cells was then represented by the ratio of the reduced area over time.

### Transwell assay

Approximately 5 × 10^4^ cells were suspended in 100 μL serum-free culture medium and added to the bottom of the transwell chambers coated with Matrigel matrix gel (Corning, USA). These chambers were then placed in a 24-well plate, with each well containing 500 μL complete culture medium. After 24 h, the cells were stained using the Wright-Giemsa Stain Kit (Nanjing Jiancheng Bio, China). Finally, images were captured using an inverted microscope. All images were captured at a magnification of 100x. In each independent experiment, we observed three distinct regions.

### RNA extraction library construction and sequencing

The RNA libraries were sequenced on the illumina NovaseqTM 6000 platform by LC Bio Technology CO.,Ltd (Hangzhou, China). We aligned reads from all samples to the homo species reference genome using HISAT2 (https://daehwankimlab.github.io/hisat2/, version: 2.2.1). This package initially filters reads based on quality information and then maps them to the reference genome. The mapped reads for each sample were assembled using StringTie (http://ccb.jhu.edu/software/stringtie/, version: 2.1.6). Subsequently, we merged all transcriptomes to reconstruct a comprehensive transcriptome using gffcompare (http://ccb.jhu.edu/software/stringtie/gffcompare.shtml, version: 0.9.8). Finally, we estimated expression levels for all transcripts and calculated FPKM values for mRNAs using StringTie and ballgown (http://www.bioconductor.org/packages/release/bioc/html/ballgown.html). Differential expression analysis of genes was conducted using DESeq2 software for two distinct groups and edgeR for two specific samples. Genes meeting the criteria of a false discovery rate (FDR) below 0.05 and an absolute fold change ≥ 2 were identified as differentially expressed [33, 34]. The heatmap of differentially expressed genes was displayed using the “pheatmap” package in R software. Subsequently, these differentially expressed genes underwent enrichment analysis for Gene Ontology (GO) functions and Kyoto Encyclopedia of Genes and Genomes (KEGG) pathways [35] by using “ClusterProfiler” package and “enrichplot” package in R software. We entered the previously obtained DEGs into the STRING platform (v12.0, https://cn.string-db.org/) [36]. Next, we screened the protein–protein interaction (PPI) pairs with a confidence score exceeding 400. Subsequently, we imported the resulting network into Cytoscape (Version 3.8.2) for additional visualization and adjustments.

### Dual-luciferase reporter assay

The Dual Luciferase Reporter Gene Assay Kit (YEASEN, China) was employed for the detection of the dual-luciferase reporter gene. We constructed a plasmid, pGL4.17, containing the IL6 promoter region. Subsequently, the plasmid and Renilla were co-transfected into the cells. After 48 h of transfection, the cells were washed with phosphate-buffered saline, lysed, and the cell lysate was collected. Finally, the collected lysate was subjected to the Firefly luciferase reaction and Renilla luciferase reaction by adding corresponding reagents, and the luminescence signals were measured using a multifunctional plate reader (ThermoFisher, USA). Utilizing Renilla luciferase as an internal reference, we assessed the activation levels of the reporter genes by comparing the ratio of Firefly to Renilla luminescence.

### Animal experiment

Twenty-four male BALB/c nude mice (6–8 weeks old) were obtained from the Institute of Zoology, Chinese Academy of Sciences in Shanghai. The animal experiments were approved by the Ethics Committee of the Laboratory Animal Center at Suzhou Medical College. Twelve of the mice were randomly divided into two groups, with subcutaneous injection of approximately 5.0 × 10^6^ NC or IFI16 786-O cells on the right dorsal side to establish a transplantation model. After 6 weeks, the mice were euthanized, and tumor volume and body weight were recorded. The remaining 12 mice were also randomly divided into two groups and injected into the mouse circulation through the tail vein (approximately 2 × 10^6^ NC or IFI16 786-O cells per mouse) to induce lung metastasis. After 6 weeks, the mice were anesthetized with isoflurane, and lung perfusion was performed with PBS. Lung tissues were then removed, fixed with Bouin's Fixative Solution (Phygene, China), and photographed to observe lung metastatic nodules.

### Statistical analysis

Independent t-test was employed to compare the continuous variables between the two groups and the χ^2^ test was used for analyzing classified data. The gene expression between the normal and tumor samples of KIRC was compared via single-factor analysis of variance. The differences of clinical characteristics between the two groups were compared with Pearson chi-square test. Kaplan–Meier survival curves were plotted by univariate survival analysis, and the log-rank test was employed to test the significance of those results. Two-tailed p < 0.05 was regarded as statistically significant. Analysis was conducted using R (version 4.2.2), SPSS (version 26) and GraphPad Prism (version 9.0.0) software. All the experiments were independently performed at least three times.

## Results

### IFI16 was highly expressed in ccRCC and associated with poor prognosis

**Fig. 1:**
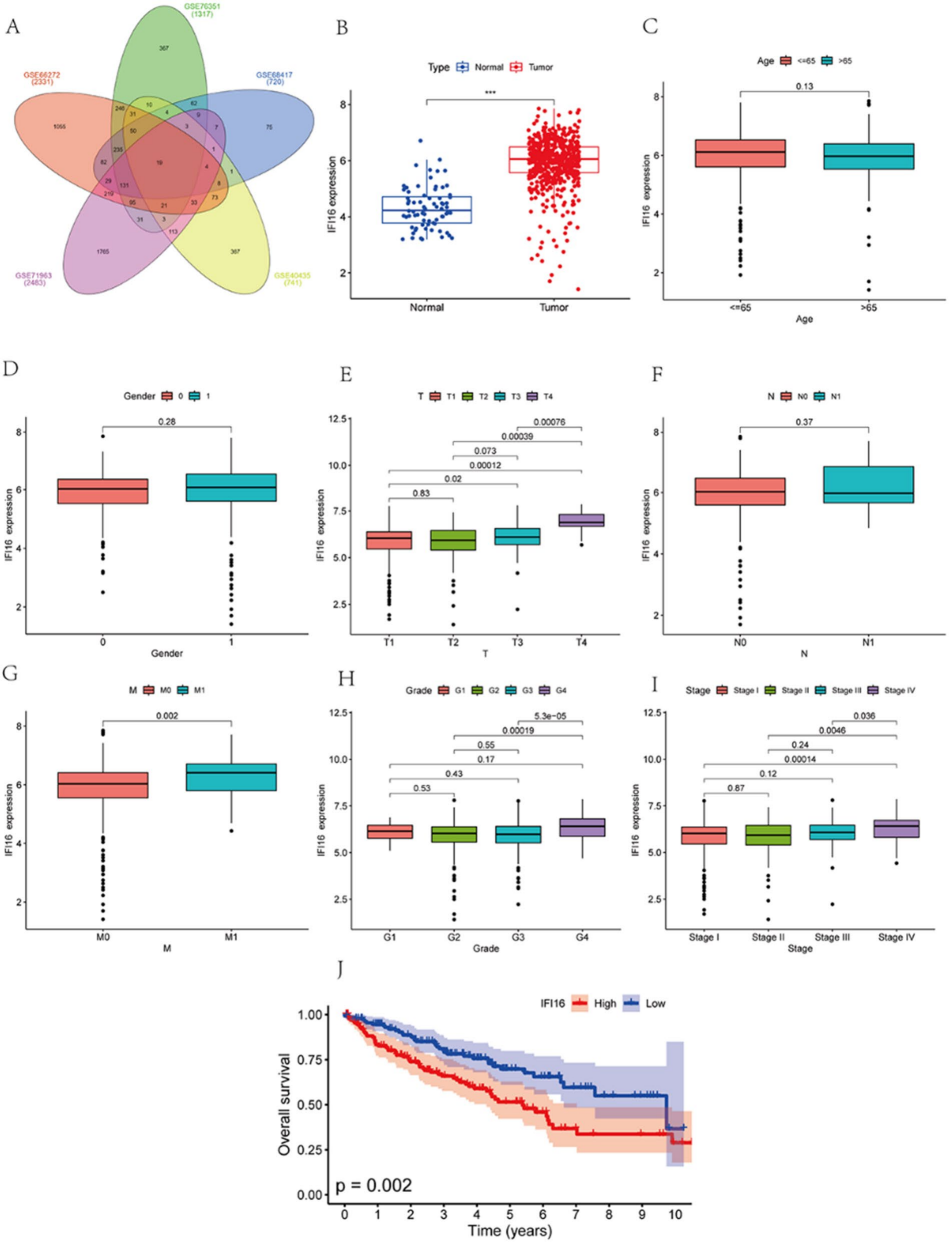
IFI16 exhibits high expression in ccRCC according to the GEO and TCGA databases. **A** An analysis of differentially expressed genes in the GEO datasets GSE66272, GSE76351, GSE68417, GSE40435, and GSE71963 was performed using a Venn diagram. **B** The differential expression of IFI16 between cancer tissue and adjacent normal tissue in the TCGA KIRC dataset. **C**–**I** In the TCGA KIRC dataset, we analyzed the expression of IFI16 among patients of different ages (**C**), genders (**D**), T stages (**E**), N stages (**F**), M stages (**G**), grade (**H**) and stage (**I**). **J** The Kaplan-Meier survival curve indicated a negative correlation between the expression of IFI16 and the survival of ccRCC patients in the TCGA KIRC dataset. The numbers on the pairwise comparisons in the figure represent p-values

First, we searched for data on clear cell renal cell carcinoma (ccRCC) in the GEO database and identified five datasets, namely GSE66272, GSE76351, GSE68417, GSE40435, and GSE71963, each containing data for both ccRCC and corresponding normal tissues. Using the online GEO2R software, we conducted comparative analyses between tumor and normal tissues to identify differentially expressed genes. Subsequently, we performed an intersection analysis among the upregulated differentially expressed genes across the five datasets, revealing 19 common genes (Fig. 1A, Additional file 1). Among these, we selected IFI16 for further investigation.

We initially validated the expression of IFI16 in ccRCC using TCGA database. Our analysis revealed that IFI16 expression was significantly higher in ccRCC compared to adjacent normal tissues (Fig. 1B). There were no statistically significant differences in IFI16 expression among patients of different ages or genders (Fig. 1C, D). Although there was no statistically significant difference in IFI16 expression among patients with different N stages, significant differences were observed in T and M stages. Moreover, higher expression of IFI16 was closely associated with higher grades and stages (Fig. 1E–I). Further survival analysis hinted at an adverse prognosis associated with elevated IFI16 expression (Fig. 1J).

**Fig. 2:**
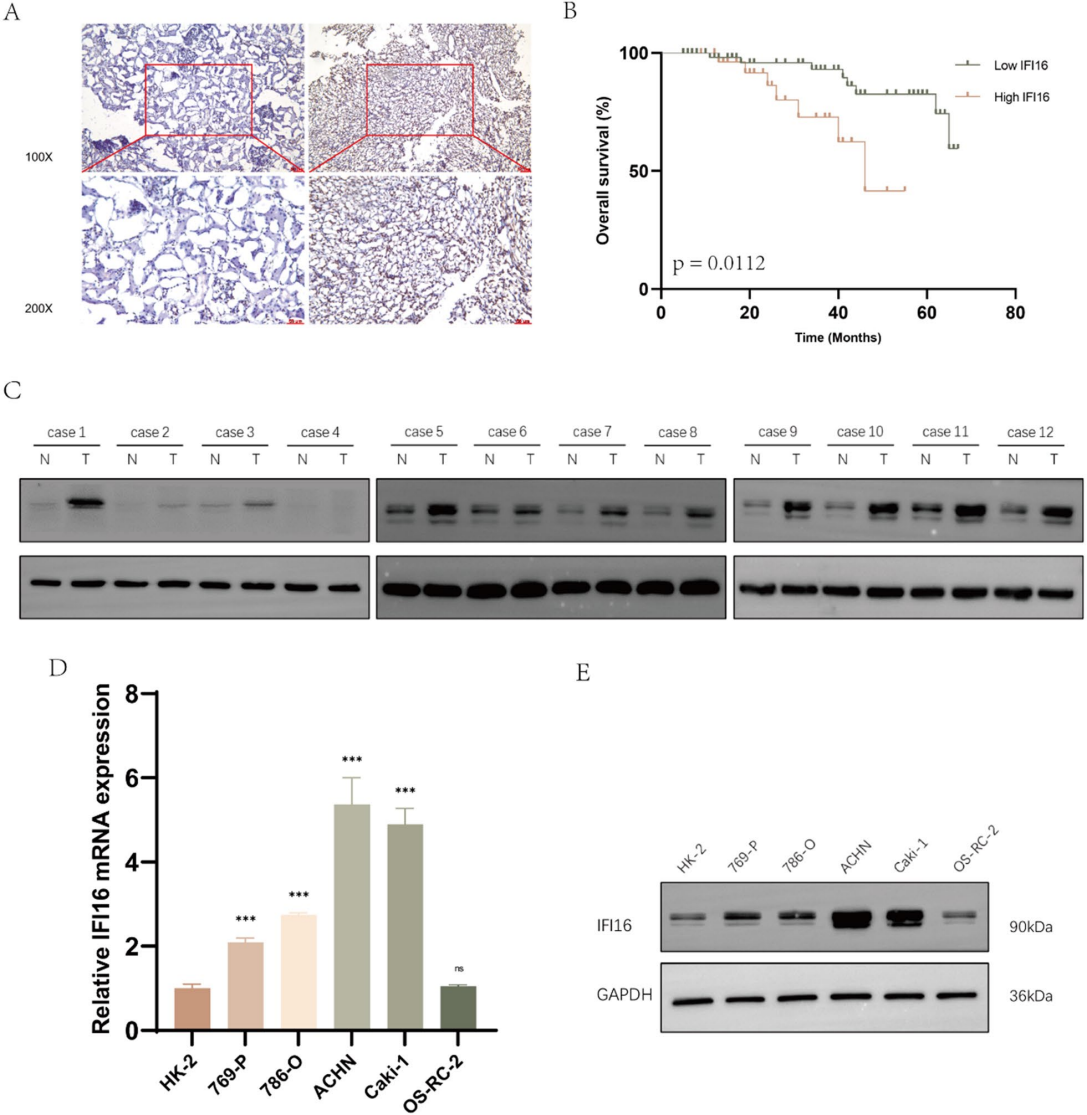
IFI16 is highly expressed in both ccRCC tissues and cell lines. **A** The representative images of IHC staining for IFI16 in cancer tissues and adjacent normal tissues from 92 pairs of ccRCC patients. **B** The overall survival of patients with high or low expression of IFI16. **C** Western blotting analysis was conducted on cancer tissues and adjacent tissues from 12 ccRCC patients. “N” represents the normal tissue while “T” refers to the tumor tissue. **D**, **E** The transcriptional and translational levels of IFI16 expression in HK-2 and ccRCC cell lines. ***p < 0.001, ns *p* > 0.05

To further assess the expression of IFI16 in ccRCC, we performed IHC on cancer tissues and paired adjacent normal tissues from 92 ccRCC patients collected at our institution. IHC staining revealed a significant increase in IFI16 expression in ccRCC tissues compared to normal tissues (Fig. 2A). This result was further confirmed by western blotting experiments (Fig. 2C). Based on the IHC results, we categorized patients into high and low IFI16 expression groups and analyzed the relationship between IFI16 expression and clinical pathological features (Table 1). Survival analysis based on patient survival data also confirmed the correlation between high IFI16 expression and a worse prognosis (Fig. 2B).

**Table 1:** Relationship between IFI16 expression and clinical pathological features in 92 patients with ccRCC

Clinicopathological features	IFI16 expression			χ^2^	*P* value
	Total	Low	High		
	92	57 (62.0%)	35 (38.0%)		
Age				0.727	0.394
< 65	42	28 (49.1)	14 (40.0)		
≥ 65	50	29 (50.9)	21 (60.0)		
Gender				2.533	0.112
Male	59	33 (57.9)	26 (74.3)		
Female	33	24 (42.1)	9 (25.7)		
TNM stage (AJCC)				6.323	0.012
I–II	57	41 (71.9)	16 (45.7)		
III–IV	35	16 (28.1)	19 (54.3)		
Fuhrman grade				12.451	< 0.001
I–II	60	45 (78.9)	15 (42.9)		
III–IV	32	12 (21.1)	20 (57.1)		

*p* value obtained through Chi-square test

Compared to the normal renal tubular epithelial cell line HK-2, RT-qPCR and western blotting experiments revealed that, at both the transcriptional and translational levels, ccRCC cell lines, except for the OS-RC-2 cell line, the 769-P, 786-O, ACHN, and Caki-1 cell lines exhibited significantly elevated expression of IFI16 (Fig. 2D, E).

### IFI16 promoted the proliferation of ccRCC cells in vitro

**Fig. 3:**
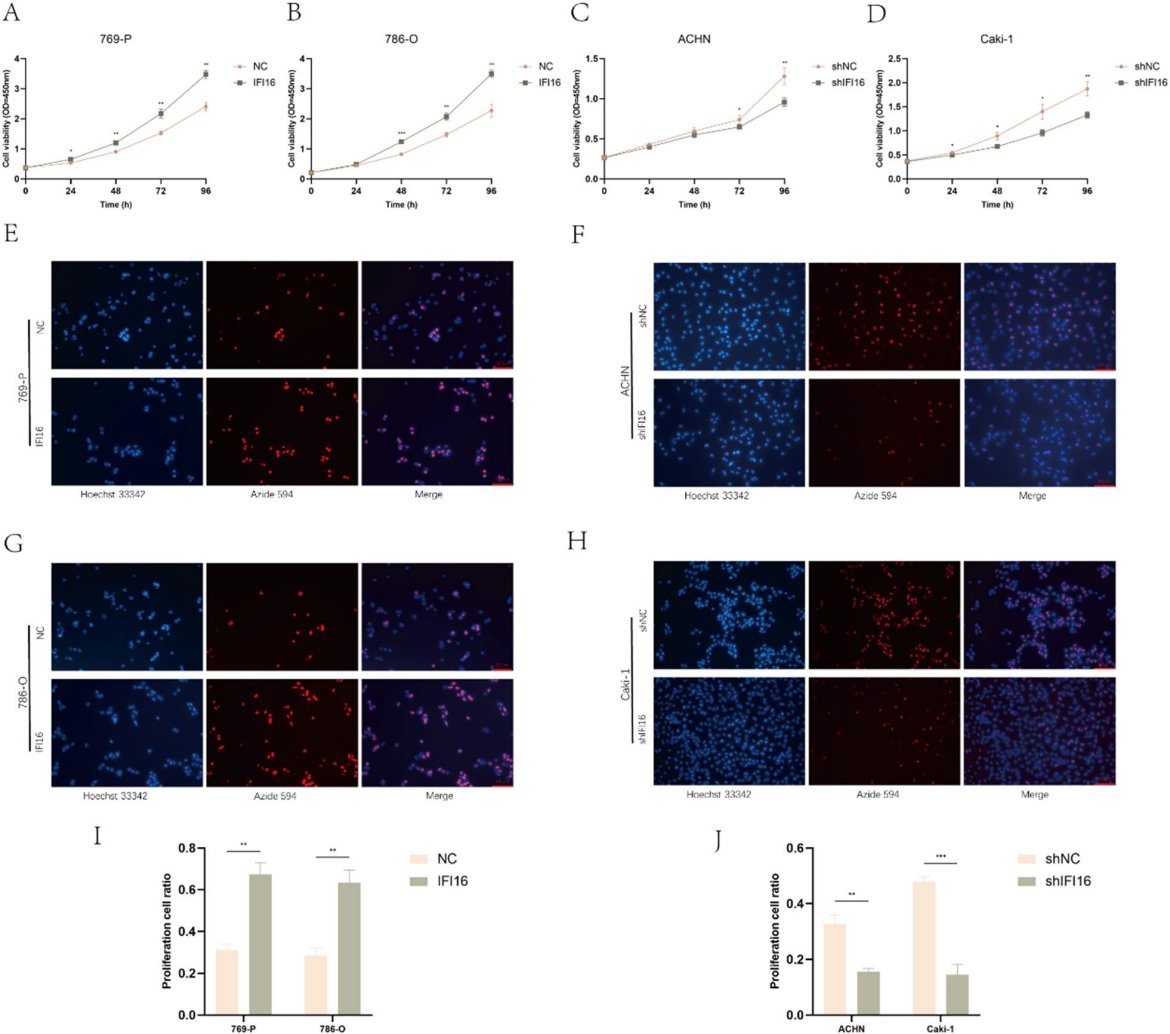
IFI16 promoted the proliferation of ccRCC cells. **A**–**D** The CCK-8 assay revealed the proliferation capacity of cells after infection with the respective lentivirus. **E**–**J** The EDU assay demonstrated the proliferation capacity of cells after overexpressing or knocking down IFI16 (approximately 200,000 cells). All experiments were performed in triplicate. Data were represented as mean ± SEM. *p < 0.05, **p < 0.01, ***p < 0.001

In order to explore the biological function of IFI16 in ccRCC, we established stable cell lines with IFI16 overexpression (769-P and 786-O) and knocked down expression (ACHN and Caki-1). We validated the efficiency of lentivirus infection through RT-qPCR and western blotting experiments, ensuring high or low expression of IFI16 at the transcription and translation levels (Fig. S1A–D). Subsequently, we selected the shIFI16-1 in ACHN and shIFI16-2 in Caki-1 cell lines with lower IFI16 expression for further experiments. CCK-8 assays revealed that overexpression of IFI16 significantly increased the proliferation activity of 769-P and 786-O cells compared to the control group (Fig. 3A, B). Conversely, knockdown of IFI16 suppressed the proliferation activity of ACHN and Caki-1 cells compared to the control group (Fig. 3C, D). EDU experiments confirmed these findings (Fig. 3E–J). In addition, we also confirmed that overexpression of IFI16 promoted the proliferation of HK-2 cells, while knocking down IFI16 reduced its proliferation ability (Fig. S2A–C, F–H).

### IFI16 promoted the migration and invasion of ccRCC cells and induced EMT

**Fig. 4:**
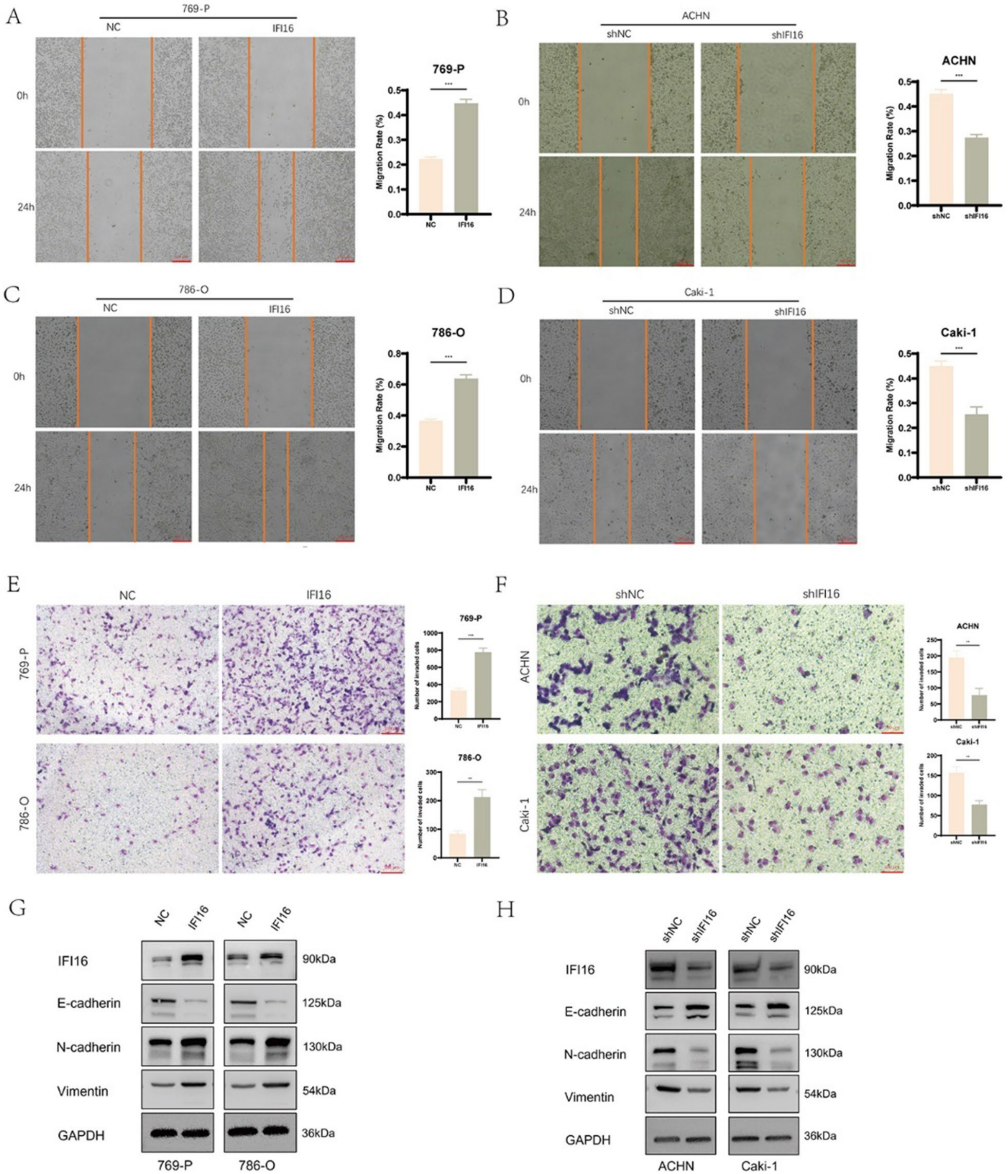
IFI16 promoted the migration and invasion of ccRCC cells and induced EMT. **A**–**D** The impact of IFI16 overexpression or knockdown on the migration capacity of ccRCC cells. **E**, **F** The impact of IFI16 overexpression or knockdown on the invasive capacity of ccRCC cells. **G**, **H** The impact of IFI16 overexpression or knockdown on the expression of EMT-related markers in ccRCC cells. All experiments were performed in triplicate. Data were represented as mean ± SEM. **p < 0.01, ***p < 0.001

We employed scratch assay and transwell assay to investigate the impact of IFI16 on the migration and invasiveness of ccRCC cells. The scratch assay revealed that overexpression of IFI16 enhanced the migratory capacity of 769-P and 786-O cells (Fig. 4A, C), while knockdown of IFI16 reduced the migration ability of ACHN and Caki-1 cells (Fig. 4B, D). Similarly, both overexpression and knockdown of IFI16 modulated the invasive potential of ccRCC cells (Fig. 4E, F). Besides, we also confirmed that overexpression of IFI16 promoted the migration and invasion abilities of HK-2 cells, while knocking down IFI16 reduced these abilities (Fig. S2D, E, I, J). To further explore whether IFI16 could promote epithelial-mesenchymal transition (EMT), we analyzed the expression of EMT-related markers in different treatment groups using western blotting. In 769-P and 786-O cells, overexpression of IFI16 led to decreased expression of E-cadherin, while N-cadherin and Vimentin expression significantly increased (Fig. 4G). Conversely, knockdown of IFI16 in ACHN and Caki-1 cells resulted in elevated E-cadherin expression and decreased N-cadherin and Vimentin expression (Fig. 4H). These findings strongly suggest the role of IFI16 in inducing EMT in ccRCC.

### IFI16 enhanced the expression of IL6 and activated the PI3K/AKT pathway

**Fig. 5:**
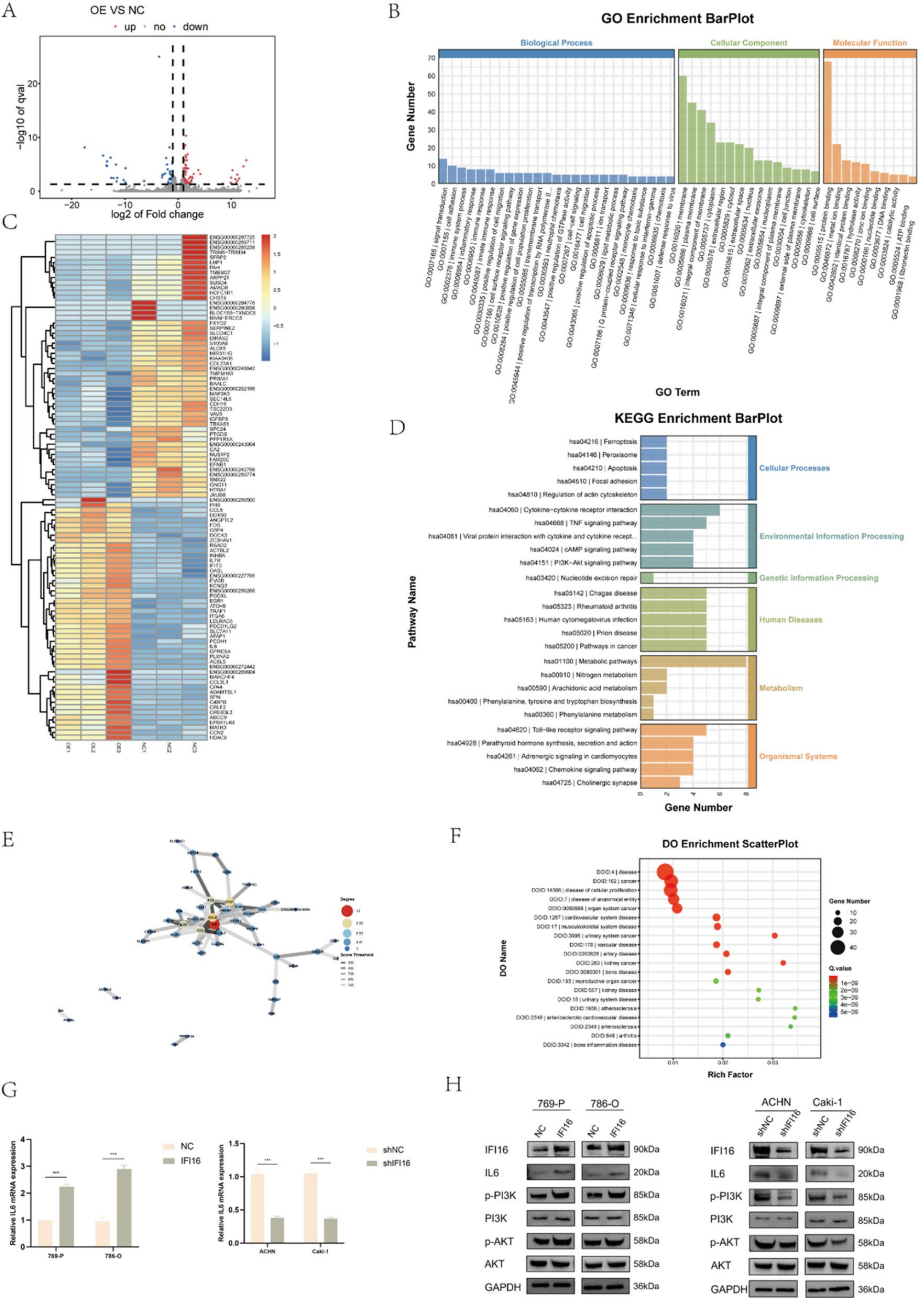
IFI16 increased IL6 expression and activated PI3K/AKT pathway. **A** The volcano plot depicted the differential transcript expression analysis of RNA-seq in 786-O cell samples between IFI16 and the NC groups. **B** Enrichment analysis of Gene Ontology (GO) based on RNA-seq assays for the gene set. **C** Heatmap depicting the top 100 Differentially Expressed Genes (DEGs). **D** Kyoto Encyclopedia of Genes and Genomes (KEGG) enrichment analysis conducted on the gene set using RNA-seq assays. **E** Conducting differential gene-protein interaction network analysis using the interaction relationships from the STRING protein interaction database. **F** Enrichment analysis of Disease Ontology (DO) based on RNA-seq assays for the gene set. **G** The mRNA levels of IL-6 after overexpression or knockdown of IFI16. **H** Western blotting revealed alterations in the expression of IL6, biomarkers of EMT and PI3K/AKT proteins following overexpression or knockdown of IFI16. ** *p* < 0.01, *** *p* < 0.001

To investigate the mechanism by which IFI16 mediates the progression of ccRCC and EMT, we conducted transcriptome sequencing and bioinformatic analysis on cell samples from the NC group and the IFI16 overexpression group. The sequencing revealed that upon IFI16 overexpression, 63 genes were upregulated, while 51 genes were downregulated (Fig. 5A). The top 100 DEGs were visualized on a heatmap (Fig. 5C). Subsequently, we performed GO, KEGG, and DO enrichment analyses on these DEGs. GO enrichment analysis indicated that this gene set is closely associated with processes such as cell adhesion, positive regulation of cell migration, positive regulation of cell population proliferation, cell migration, and extracellular exosome (Fig. 5B, Fig.S3). The KEGG enrichment analysis indicated that, following activation by IFI16, the PI3K/AKT signaling pathway might be involved in the progression of ccRCC (Fig. 5D). DO enrichment analysis further confirmed enrichment in kidney cancer-related genes (Fig. 5F). To identify potential downstream molecules regulated by IFI16, we conducted differential gene-protein interaction network analysis using the STRING protein interaction database and constructed a network diagram. The analysis revealed that IL6 was the most important node in the network (Fig. 5E). RT-qPCR and western blotting experiments also confirmed a correlation between the expression trend of IL6 and IFI16 in cells (Fig. 5G, H). Additional, overexpression of IFI16 led to the increasement of p-PI3K and p-AKT, whereas knockdown of IFI16 resulted in the reduction of p-PI3K and p-AKT (Fig. 5H).

RNA sequencing data indicated that IFI16 increased the expression of IL6 at the transcriptional level. Western blotting experiments confirmed changes in IL6 at the translational level. Consequently, we hypothesize that IFI16 can activate IL6 transcription. To test our hypothesis, we initially constructed a luciferase reporter gene containing the IL6 promoter region. The results revealed enhanced transcription of IL6 upon IFI16 overexpression (Fig. S1E). Conversely, knocking down IFI16 led to decreased IL6 transcription (Fig. S1F). These findings suggest that IFI16 can activate IL6 transcription and induce the PI3K/AKT signaling pathway by upregulating the transcription and translation of IL6.

### IL6 promoted the proliferation, migration, and invasion of ccRCC cells in vitro

**Fig. 6:**
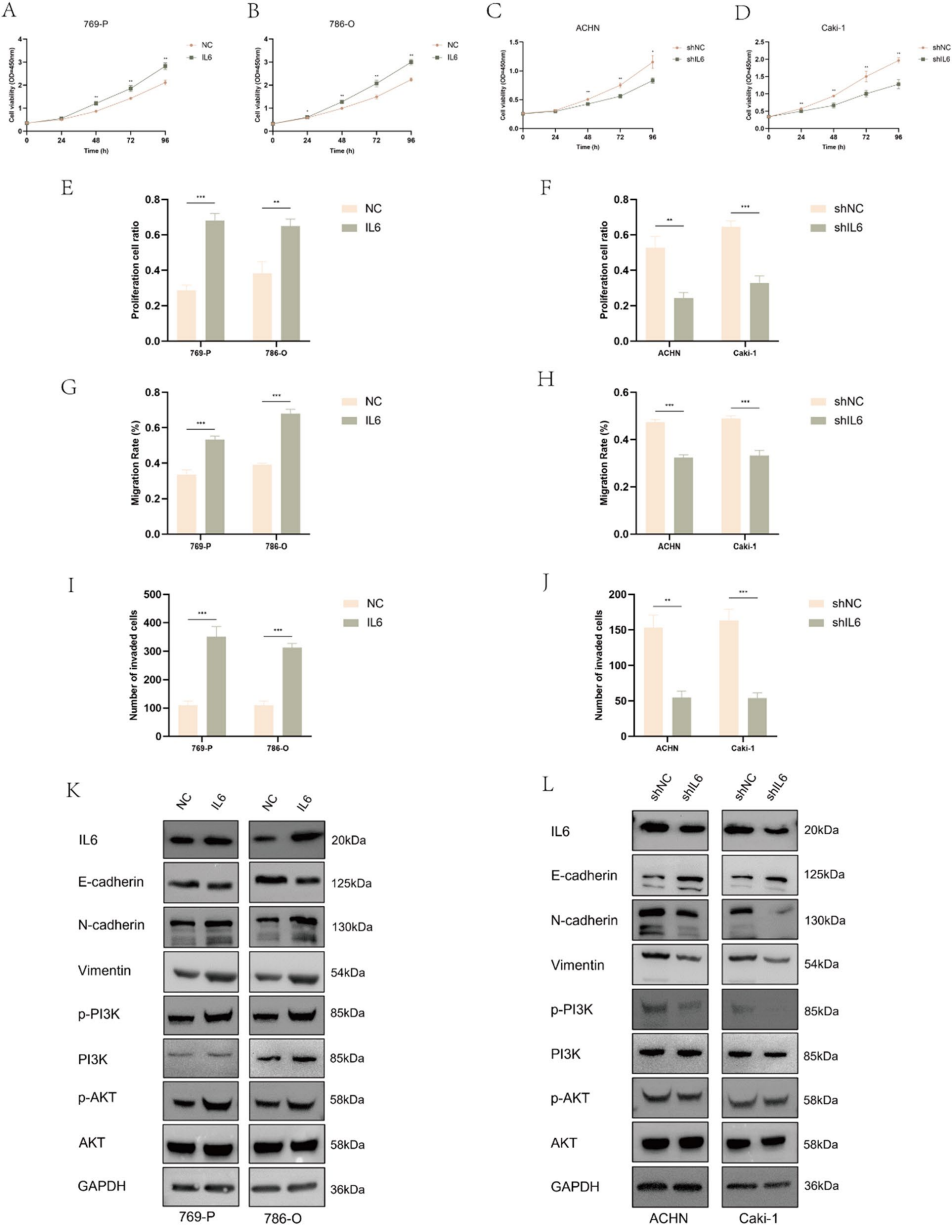
IL6 promoted the proliferation, migration, and invasion of ccRCC cells in vitro. **A**–**D** The CCK-8 assay revealed the proliferation capacity of cells after infection with the respective lentivirus. **E**, **F** The EDU assay demonstrated the proliferation capacity of cells after IL6 overexpression or knockdown. **G**, **H** The impact of IL6 overexpression or knockdown on the migration capacity of ccRCC cells. **I**, **J** The impact of IL6 overexpression or knockdown on the invasive capacity of ccRCC cells. **K**, **L** Overexpression or knockdown of IL6 in ccRCC cells affected the expression of markers related to EMT and the PI3K/AKT pathway. All experiments were performed in triplicate. Data were represented as mean ± SEM. *p < 0.05, **p < 0.01, ***p < 0.001

To investigate the impact of IL6 on ccRCC cells, we initially established stable cell lines overexpressing and knocking down IL6. We then validated these cell lines at the transcriptional and translational levels (Fig. S1G–J). In the IL6 knockdown groups of ACHN and Caki-1 cells, the shIL6-1 subgroup consistently exhibited higher efficiency, prompting us to select it for subsequent experiments. The CCK-8 assay revealed that overexpression of IL6 significantly increased the proliferation rate of 769-P and 786-O cells (Fig. 6A, B). Conversely, knockdown of IL6 suppressed the proliferation rate of ACHN and Caki-1 cells (Fig. 6C, D). This observation was further confirmed by the EdU assay (Fig. 6E, F, Fig. S4A–D). Furthermore, the scratch assay demonstrated that IL6 overexpression enhanced the migratory capacity of 769-P and 786-O cells (Fig. 6G, Fig. S4E), while IL6 knockdown reduced the migration ability of ACHN and Caki-1 cells (Fig. 6H, Fig. S4F). Similarly, the transwell assay underscored the consistent impact of IL6 on cell invasion (Fig. 6I, J, Fig. S4G, H). Finally, western blotting confirmed the association between IL6 and the PI3K/AKT signaling pathway as well as EMT. IL6 overexpression led to decreased E-cadherin expression in 769-P and 786-O cells, while N-cadherin and Vimentin expression significantly increased (Fig. 6K). Additionally, levels of p-PI3K and p-AKT were markedly elevated (Fig. 6K). Conversely, knocking down IL6 in ACHN and Caki-1 cells produced opposite results (Fig. 6L).

### The IL6/PI3K/AKT axis was essential for IFI16-mediated ccRCC proliferation and progression

**Fig. 7:**
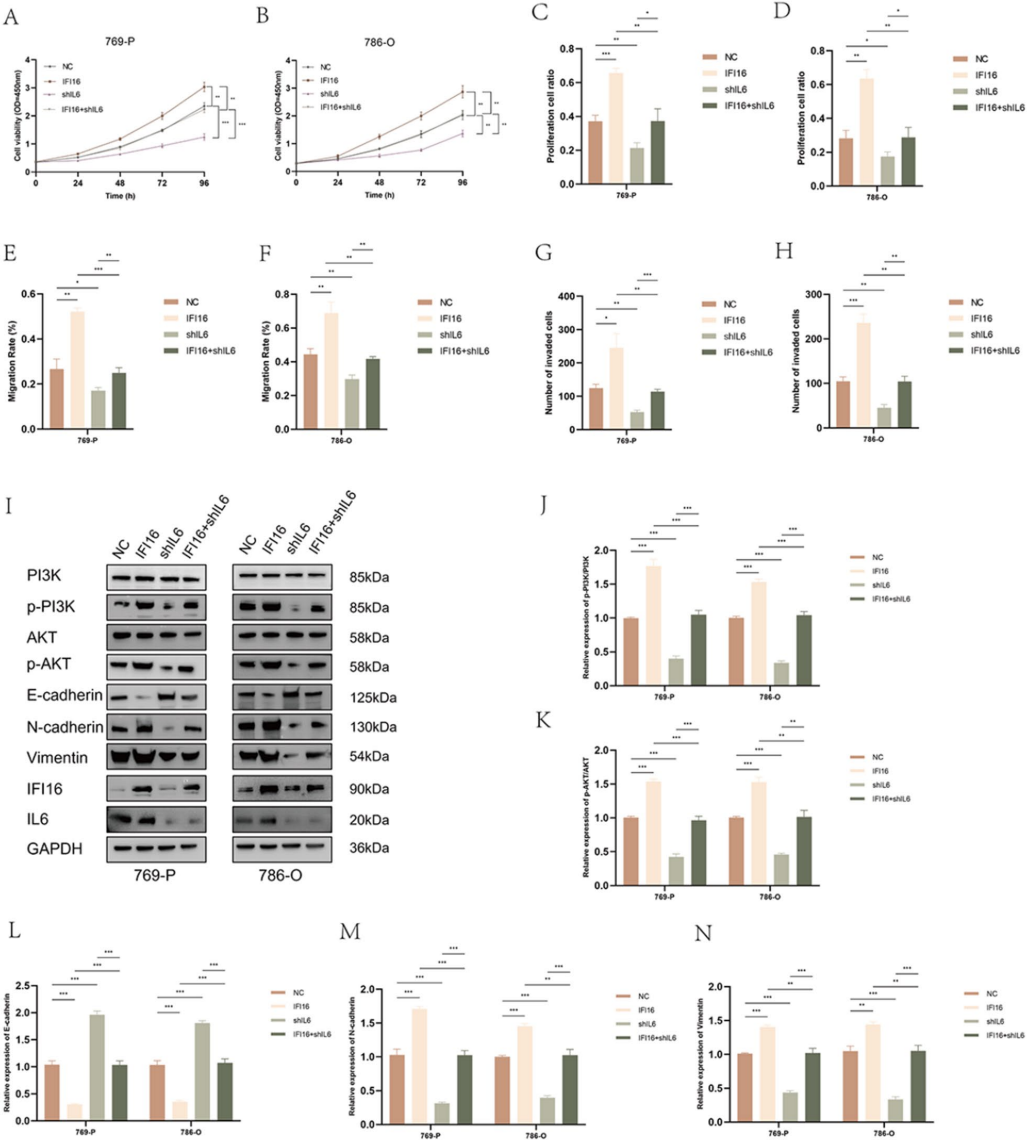
Knocking down IL6 inhibited the role of IFI16 in promoting ccRCC progression. **A**, **B** The CCK-8 assay demonstrated the proliferative capacity of cells solely overexpressing IFI16, knocking down IL6, and both overexpressing IFI16 and knocking down IL6. **C**, **D** The EDU assay demonstrated the proliferation capacity of cells after solely overexpressing IFI16, knocking down IL6, and both overexpressing IFI16 and knocking down IL6. **E**, **F** The impact of solely overexpressing IFI16, knocking down IL6, and both overexpressing IFI16 and knocking down IL6 on the migration capacity of ccRCC cells. **G**, **H** The impact of solely overexpressing IFI16, knocking down IL6, and both overexpressing IFI16 and knocking down IL6 on the invasive capacity of ccRCC cells. **I**–**N** Western blotting revealed the expression of the indicated proteins after solely overexpressing IFI16, knocking down IL6, and both overexpressing IFI16 and knocking down IL6. All experiments were performed in triplicate. Data were represented as mean ± SEM. *p < 0.05, **p < 0.01, ***p < 0.001

To further investigate the epigenetic regulatory role of IL6 in IFI16-mediated effects on ccRCC, we conducted rescue experiments by knocking down IL6 in 769-P-IFI16 and 786-O-IFI16 cells. The CCK-8 assay demonstrated that knocking down IL6 could counteract the impact of IFI16 overexpression on the proliferation rate of 769-P and 786-O cells (Fig. 7A, B). Similarly, the EdU assay yielded consistent results (Fig. 7C, D, Fig. S5A, B). Moreover, the scratch assay and transwell assay indicated that knocking down IL6 could also mitigate the enhancement of migration and invasion abilities induced by IFI16 overexpression in 769-P and 786-O cells (Fig. 7E–H, Fig. S5C–E). Western blotting revealed that IFI16 overexpression significantly increased the phosphorylation levels of PI3K and AKT in cells, while knocking down IL6 markedly reduced the phosphorylation levels of PI3K and AKT. Interestingly, simultaneous overexpression of IFI16 and knocking down IL6 restored the phosphorylation levels of PI3K and AKT to normal (Fig. 7I–K). Additionally, the impact of IFI16 overexpression or IL6 knockdown on the expression of EMT markers was consistent with previous findings. Notably, when IFI16 overexpression and IL6 knockdown were combined, the expression of E-cadherin, N-cadherin, and Vimentin in 769-P and 786-O cells could be reversed (Fig. 7I, L–N). These experiments collectively demonstrate that knocking down IL6 can reverse the enhanced effects of IFI16 overexpression on ccRCC proliferation, migration, invasion, EMT, and the PI3K/AKT signaling pathway.

**Fig. 8:**
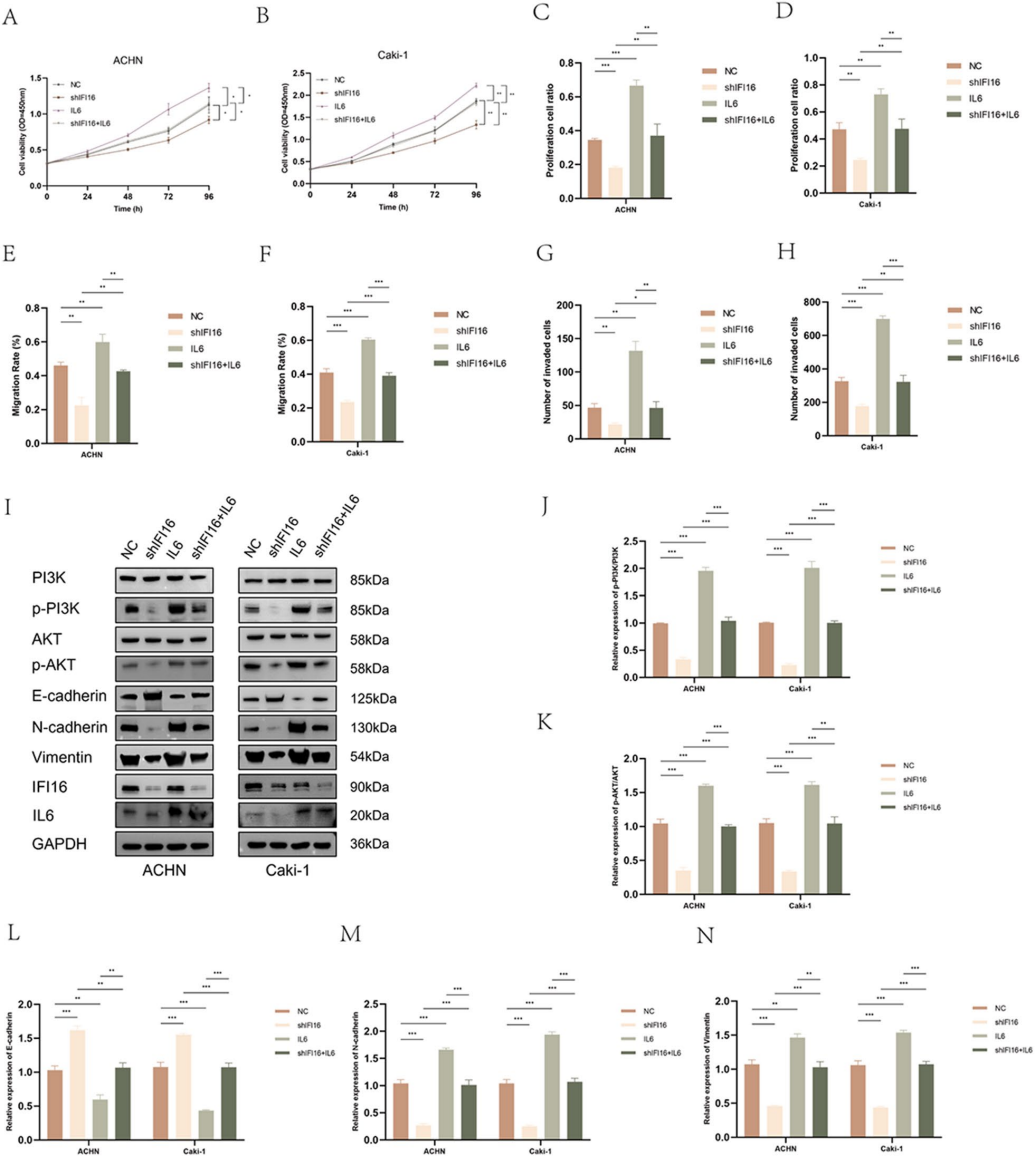
Overexpression of IL6 reversed the inhibitory effect of knocking down IFI16 on ccRCC progression. **A**, **B** The CCK-8 assay demonstrated the proliferative capacity of cells solely knocking down IFI16, overexpressing IL6, and both knocking down IFI16 and overexpressing IL6. **C**, **D** The EDU assay demonstrated the proliferation capacity of cells after solely knocking down IFI16, overexpressing IL6, and both knocking down IFI16 and overexpressing IL6. **E**, **F** The impact of solely knocking down IFI16, overexpressing IL6, and both knocking down IFI16 and overexpressing IL6 on the migration capacity of ccRCC cells. **G**, **H** The impact of solely knocking down IFI16, overexpressing IL6, and both knocking down IFI16 and overexpressing IL6 on the invasive capacity of ccRCC cells. **I**–**N** Western blotting revealed the expression of the indicated proteins after solely knocking down IFI16, overexpressing IL6, and both knocking down IFI16 and overexpressing IL6. All experiments were performed in triplicate. Data were represented as mean ± SEM. *p < 0.05, **p < 0.01, ***p < 0.001

Similarly, we conducted rescue experiments by overexpressing IL6 in ACHN-shIFI16 and Caki-1-shIFI16 cells. The CCK-8 assay revealed that IL6 overexpression could counteract the impact of IFI16 knockdown on the proliferation rate of ACHN and Caki-1 cells (Fig. 8A, B). Consistent results were obtained from the EdU assay (Fig. 8C, D, Fig. S5F, G). Furthermore, the scratch assay and transwell assay demonstrated that IL6 overexpression could also reverse the weakened migration and invasion abilities induced by IFI16 knockdown in ACHN and Caki-1 cells (Fig. 8E–H, Fig. S5H–J). Western blotting confirmed that IFI16 knockdown significantly reduced the phosphorylation levels of PI3K and AKT in cells, while IL6 overexpression markedly increased their phosphorylation levels. Remarkably, simultaneous IFI16 knockdown and IL6 overexpression restored the phosphorylation levels of PI3K and AKT to normal (Fig. 8I–K). Similar to the previous observations, the impact of IFI16 knockdown or IL6 overexpression on the expression of EMT markers was consistent. Notably, when IFI16 knockdown and IL6 overexpression were combined, the expression of E-cadherin, N-cadherin, and Vimentin in ACHN and Caki-1 cells could be reversed (Fig. 8I, L–N). These experiments suggest that IL6 / PI3K / AKT axis plays a crucial role in IFI16-induced effects on ccRCC cell proliferation, migration, and invasion.

### IFI16 promoted tumor occurrence and metastasis of ccRCC in vivo

**Fig. 9:**
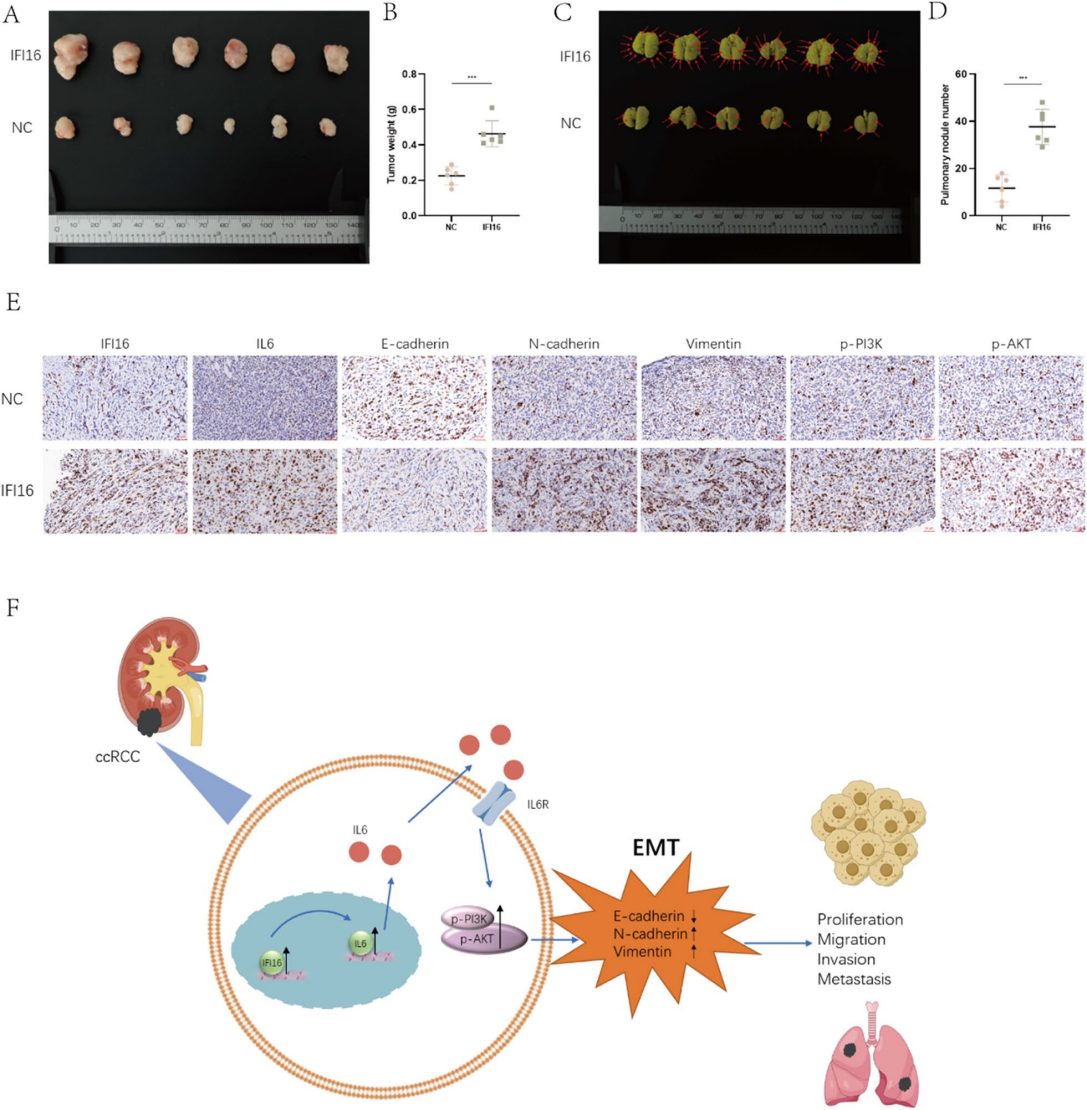
IFI16 promoted tumor occurrence and metastasis of ccRCC in vivo. **A**, **B** Subcutaneous injection of 786-O-NC or 786-O-IFI16 cells into the flank of BALB/c nude mice established xenograft models and allowed for the weighing of subcutaneous tumors (n = 6 each group). **C**, **D** Injected the same quantity of 786-O-NC or 786-O-IFI16 cells via tail vein into BALB/c nude mice. After 6 weeks, euthanized the mice and visually observed pulmonary metastatic nodules (n = 6 each group). **E** IHC staining of subcutaneous tumors. **F** The mechanism graph of the regulatory network and function of IFI16 in ccRCC. ***p < 0.001

Finally, we established an in vivo model to determine whether IFI16 played a role in the proliferation and metastasis of ccRCC cells within the body. We subcutaneously injected an equal number of 786-O-NC or 786-O-IFI16 cells into the dorsal subcutaneous tissue of BALB/c nude mice to create a xenograft tumor model. Simultaneously, we established a lung metastasis model in the remaining mice by intravenously injecting the same number of 786-O-NC or 786-O-IFI16 cells. After euthanizing the mice, we retrieved tumor tissue for analysis. The results indicated that the tumor volume and weight were significantly higher in the IFI16 overexpression group compared to the control group (Fig. 9A, B). Additionally, we observed a markedly higher number and size of lung metastatic nodules in the IFI16 overexpression group compared to the control group (Fig. 9C, D). We performed IHC staining on subcutaneous tumor specimens from nude mice. The results revealed that after overexpressing IFI16, the levels of IL6, N-cadherin, Vimentin, p-PI3K, and p-AKT were significantly elevated, while E-cadherin levels were markedly suppressed (Fig. 9E). Therefore, we have reason to believe that IFI16 plays a crucial role in ccRCC tumor occurrence and metastasis within the body.

In summary, IFI16 exerts its effects by modulating IL6, which activates the IL6/PI3K/AKT pathway, promoting the occurrence of ccRCC EMT, and ultimately enhancing tumor proliferation and metastasis (Fig. 9F).

## Discussion

For the treatment of advanced RCC patients, although there have been continuous advancements, truly effective therapeutic approaches remain elusive [37]. Prior to 2005, interferon-α and high-dose interleukin-2 were the only treatments proven effective in a small subset of patients [38]. As research on angiogenesis in tumor development progressed, treatment strategies targeting the vascular endothelial growth factor (VEGF) pathway emerged, along with a series of other approaches such as targeting the mammalian target of mTOR pathway (rapamycin) and immune checkpoint blockers [39]. Given that ccRCC is the most common pathological type within RCC, naturally, our research focuses on targeted therapies for ccRCC. In our study, we first identified common differentially expressed genes through multiple GEO datasets and then validated them in the TCGA database. This led us to choose IFI16, a gene that has been reported for its biological functions in other tumors but has received limited attention in RCC. Subsequent analysis of clinical specimens and information from collected ccRCC patients revealed that IFI16 is indeed highly expressed in ccRCC tissues. Furthermore, elevated IFI16 expression correlates closely with higher tumor grade, stage, and poorer prognosis. Cell experiments further confirmed IFI16’s ability to promote ccRCC cell proliferation, migration, invasion, and EMT. In vivo experiments also demonstrated its role in enhancing proliferation and metastasis. Therefore, we propose that IFI16 may serve as a potential prognostic marker for ccRCC patients, especially those in advanced stages, and could be a potential therapeutic target for ccRCC patients.

As a member of the IFN-inducible protein family [40], IFI16 was previously primarily associated with amplifying DNA damage responses and accelerating cellular aging [41]. IFI16 can sense viral dsDNA and mediate IFN-I production following exposure to ionizing radiation [42, 43]. Additionally, IFI16’s recognition of HSV-1 viral DNA can mediate recruitment of IFI16-STING-dependent TANK-binding kinase (TBK1) and DEAD-box polypeptide 3 (DDX3), activation of the IRF3 and NF-κB pathways, and production of IFN-β [5]. Previous studies have indicated that IFI16 plays diverse roles in the development of various cancers [7–12]. Although Yu et al. validated the correlation between IFI16 and clinical pathological features of ccRCC using TCGA database, and confirmed the impact of IFI16 on ccRCC cell proliferation, migration, and invasion by knocking down IFI16 at the cellular level [44]. However, they did not overexpress IFI16 for positive validation and lacked subsequent mechanistic exploration and in vivo validation. Our study aims to fill this gap and shed light on the role of IFI16 in ccRCC development. We didn’t solely rely on GEO and TCGA datebase analysis, but also rely on our clinical data analysis. We further substantiated the role of IFI16 by bidirectional validation through overexpressing and knocking down IFI16 in multiple cell lines. Additionally, we made a groundbreaking discovery of IFI16’s downstream molecule, IL6, and confirmed the role of the IFI16/IL6/PI3K/AKT axis in ccRCC. This finding is highly significant for targeted therapy in ccRCC. Cytokines and cytokine receptors have been extensively studied and serve as cancer targets in cancer therapy [45]. IL6 is a multifunctional cytokine that can exert pro-inflammatory or anti-inflammatory effects. It mediates activation of the JAK/STAT3, Ras/MAPK, and PI3K/AKT signaling pathways, promoting tumor development [46]. The occurrence of EMT in RCC is significantly associated with tumor metastasis [47]. In our experiments, we discovered for the first time that IFI16 overexpression activates transcription of IL6 in dual-luciferase assays and further induces activation of the PI3K/AKT pathway and EMT in ccRCC cells. According to reports, IFI16 can directly bind to the C-terminal region of p53 and enhance p53-mediated transcriptional activation without affecting the steady-state levels of p53 [48]. Fu et al. [49] discovered that USP12 promoted IFI16-mediated innate antiviral signaling. Knockdown of USP12 impaired the activation of the IFI16-STING-IRF3/NF-κB pathway triggered by DNA viruses and the expression of downstream genes, including IL6. Thompson et al. [50] discovered that stable knockdown of IFI16 severely attenuated the type I interferon response to DNA ligands and viruses. In contrast, the expression of NF-κB-regulated cytokines IL-6 and IL-1β remained unaffected in IFI16-knockdown cells. And they predicted that IFI16 might directly bind to the promoter region of the IFN-α gene or associate with other transcription factors or co-regulators to regulate its expression. These divergent findings underscore the complexity of IFI16’s regulatory functions. Based on our experimental results, we hypothesize that in ccRCC, IFI16 may directly bind to the promoter region of IL6, thereby enhancing its transcription and translation, and activating the PI3K/AKT signaling pathway. Alternatively, IFI16 could interact with a transcription factor to further regulate IL6 expression. However, further in-depth research is needed to explore these mechanisms.

In summary, our research suggests that IFI16 is a potential biomarker for diagnosing and assessing ccRCC patient progression. To date, specific inhibitors targeting IFI16 have not been reported, but we believe that developing specific IFI16 inhibitors holds promise as a clinical therapeutic strategy for ccRCC patients.

## Conclusion

In summary, IFI16 can enhance the transcription and translation of IL6, further activating the PI3K/AKT signaling pathway and inducing EMT. Consequently, this promotes the proliferation and metastasis of ccRCC. However, it remains unclear whether IFI16 can directly activate IL6, necessitating further research.

### Supplementary Information

Additional file 1. The gene lists of Venn diagram.

Additional file 2. The primers using for RT-qPCR, the sequences of shRNAs using for gene silencing, the sequence of IL6 promoter region and the supplementary figure legends.

Additional file 3. The RNA-seq, showing the OE VS NC genes significantly differential expression.

Additional file 4. Figure S1. Validation of stable cell lines and dual-luciferase reporter assay.

Additional file 5. Figure S2. Validation of stable cell lines and supplementary cell experiments.

Additional file 6. Figure S3. Enrichment analysis of Gene Ontology.

Additional file 7. Figure S4. Typical graphs after overexpression or knockdown of IL6.

Additional file 8. Figure S5. Typical graphs of the rescue experiments.

## Data Availability

The datasets used and analysed during the current study are available from the corresponding author on reasonable request.

## Abbreviations

RCC: Renal cell carcinoma
ccRCC: Clear cell renal cell carcinoma
EMT: Epithelial-mesenchymal transition
DEGs: Differentially expressed genes
IHC: Immunohistochemistry
RT-qPCR: Reverse transcription quantitative polymerase chain reaction
GO: Gene ontology
KEGG: Kyoto encyclopedia of genes and genomes
DO: Disease ontology
