# Supplementary material for: IFI16 promotes the progression of clear cell renal cell carcinoma through the IL6/PI3K/AKT axis
Source: J Transl Med. 2024 Jun 3;22:533. doi: 10.1186/s12967-024-05354-w (PMC11149187; doi:10.1186/s12967-024-05354-w)
Supplement: Supplementary file 2 — Additional file 2. The primers using for RT-qPCR, the sequences of shRNAs using for gene silencing, the sequence of IL6 promoter region and the supplementary figure legends. [file 12967_2024_5354_MOESM2_ESM.docx]

**Supplementary Materials**

**1.The primers using for RT-qPCR**

*IFI16:*

Forward Primer:

5’ AGACTGAAGACTGAACCTGAAGA 3’

Reverse Primer:

5’ GAACCCATTGCGGCAAACATA 3’

*IL6:*

Forward Primer:

5’ ACTCACCTCTTCAGAACGAATTG 3’

Reverse Primer:

5’ CCATCTTTGGAAGGTTCAGGTTG 3’

*GAPDH:*

Forward Primer:

5’ GGAGCGAGATCCCTCCAAAAT 3’

Reverse Primer:

5’ GGCTGTTGTCATACTTCTCATGG 3’

**2.The sequences of shRNAs using for gene silencing**

*IFI16:*

sh1

Forward oligo:

5’ CCGGGTCAGGTAACTCCCAGAAGAACTCGAGTTCTTCTGGGAGTTACCTGACTTTTTG 3’

Reverse oligo:

5’ AATTCAAAAAGTCAGGTAACTCCCAGAAGAACTCGAGTTCTTCTGGGAGTTACCTGAC 3’

sh2

Forward oligo:

5’ CCGGGACAGGACAATGTCACAATATCTCGAGATATTGTGACATTGTCCTGTCTTTTTG 3’

Reverse oligo:

5’ AATTCAAAAAGACAGGACAATGTCACAATATCTCGAGATATTGTGACATTGTCCTGTC 3’

*IL6:*

sh1

Forward oligo:

5’ CCGGCAGAACGAATTGACAAACAAACTCGAGTTTGTTTGTCAATTCGTTCTGTTTTTG 3’

Reverse oligo:

5’ AATTCAAAAACAGAACGAATTGACAAACAAACTCGAGTTTGTTTGTCAATTCGTTCTG 3’

sh2

Forward oligo:

5’ CCGGGACATGTAACAAGAGTAACATCTCGAGATGTTACTCTTGTTACATGTCTTTTTG 3’

Reverse oligo:

5’ AATTCAAAAAGACATGTAACAAGAGTAACATCTCGAGATGTTACTCTTGTTACATGTC 3’

**3.The sequence of IL6 promoter region (-2000--99bp)**

GGTCCTTGATGTAACAGCCAGGATCAAACAGCTGGGAAGACGAGAAAACCTTTCCCAGGCTAGGATAACAGAGGATTTGGTTGAAAATACAGGCAATTAGGTGCTACCTCTGGGAAAAGGGGCCAGGAGAGGAAGGAGACACTTTTCCCTGCATGCCCTGATGTCCTATTTGAACATTTTATCATGAACACGAACTTCCTATTTAAAAAACACTTTTTATTGAAAAGATAAATCTGTGTGTTGTATTGTGTCACTCAGTTCAAGTACTTGAAATTTATTGAATTGTATTTTCTAAAAAATAGATAGTTGAGTAAAAGCAAGCTCACATTACATAGACGGATCACAGTGCACGGCTGCGGAGCTGGGAGCAGTGGCTTCGTTTCATGCAGGAAAGAGAACTTGGTTCAGGAGTGTCTACGTTGCTTAAGACAGGAGAGCACTAAAAATGAAACCATCCAGCCATCCTCCCCCATTTTCATTTTCACACCAAAGAATCCCACCGCGGCAGAGGACCACCGTCTCTGTTTAGACAATCGGTGAAGAATGGATGACCTCACTTTCCCCAACAGGCGGGTCCTGAAATGTTATGCACGAAACAAAACTTGAGTAAATGCCCAACAGAGGTCACTGTTTTATCGATCTTGAAGAGATCTCTTCTTAGCAAAGCAAAGAAACCGATTGTGAAGGTAACACCATGTTTGGTAAATAAGTGTTTTGGTGTTGTGCAAGGGTCTGGTTTCAGCCTGAAGCCATCTCAGAGCTGTCTGGGT

CTCTGGAGACTGGAGGGACAACCTAGTCTAGAGCCCATTTGCATGAGACCAAGGATCCTCCTGCAAGAGACACCATCCTGAGGGAAGAGGGCTTCTGAACCAGCTTGACCCAATAAGAAATTCTTGGGTGCCGACGCGGAAGCAGATTCAGAGCCTAGAGCCGTGCCTGCGTCCGTAGTTTCCTTCTAGCTTCTTTTGATTTCAAATCAAGACTTACAGGGAGAGGGAGCGATAAACACAAACTCTGCAAGATGCCACAAGGTCCTCCTTTGACATCCCCAACAAAGAGGTGAGTAGTATTCTCCCCCTTTCTGCCCTGAACCAAGTGGGCTTCAGTAATTTCAGGGCTCCAGGAGACCTGGGGCCCATGCAGGTGCCCCAGTGAAACAGTGGTGAAGAGACTCAGTGGCAATGGGGAGAGCACTGGCAGCACAAGGCAAACCTCTGGCACAGAGAGCAAAGTCCTCACTGGGAGGATTCCCAAGGGGTCACTTGGGAGAGGGCAGGGCAGCAGCCAACCTCCTCTAAGTGGGCTGAAGCAGGTGAAGAAAGTGGCAGAAGCCACGCGGTGGCAAAAAGGAGTCACACACTCCACCTGGAGACGCCTTGAAGTAACTGCACGAAATTTGA

GGATGGCCAGGCAGTTCTACAACAGCCGCTCACAGGGAGAGCCAGAACACAGAAGAACTCAGATGACTGGTAGTATTACCTTCTTCATAATCCCAGGCTTGGGGGGCTGCGATGGAGTCAGAGGAAACTCAGTTCAGAACATCTTTGGTTTTTACAAATACAAATTAACTGGAACGCTAAATTCTAGCCTGTTAATCTGGTCACTGAAAAAAAATTTTTTTTTTTTCAAAAAACATAGCTTTAGCTTATTTTTTTTCTCTTTGTAAAACTTCGTGCATGACTTCAGCTTTACTCTTTGTCAAGACATGCCAAAGTGCTGAGTCACTAATAAAAGAAAAAAAGAAAGTAAAGGAAGAGTGGTTCTGCTTCTTAGCGCTAGCCTCAATGACGACCTAAGCTGCACTTTTCCCCCTAGTTGTGTCTTGCCATGCTAAAGGACGTCACATTGCACAATCTTAATAAGGTTTCCAATCAGCCCCACCCGCTCTGGCCCCACCCTCACCCTCCAACAAAGATTTATCAAATGTGGGATTTTCCCATGAGTCTCAATATTAGAGTCTCAACCCCCAATAAATATAGGACTGGAGATGTCTGAGGCTCATTCTGCCCTCGAGCCCACCGGGAACGAAAGAGAAGCTCTATCTCCCCTCCAGGAGCCCAGCTATGAACTCCTTCTCCACAAGTAAGTGCAGGAAATCCT

**Supplementary Figure Legends**

*Supplementary Figure 1*.

**A, C** After infection with lentivirus overexpressing IFI16, both transcription and translation levels of IFI16 were assessed in 769-P and 786-O cells. **B, D** After infection with lentivirus carrying a knockdown construct for IFI16, the transcription and translation levels of IFI16 in ACHN and Caki-1 cells were assessed. **E, F** Luciferase reporter gene assays detecting IL6 promoter region activity after overexpressing or knocking down IFI16. **G, I** After infection with lentivirus overexpression of IL6, both transcription and translation levels of IL6 were assessed in 769-P and 786-O cells. **H, J** After infection with lentivirus carrying a knockdown construct for IL6, the transcription and translation levels of IL6 in ACHN and Caki-1 cells were assessed. All experiments were performed in triplicate. Data were represented as mean ± SEM.

*Supplementary Figure 2*.

**A** After infection with lentivirus overexpressing IFI16, translation level of IFI16 was assessed in HK-2 cells. **F** After infection with lentivirus carrying a knockdown construct for IFI16, translation level of IFI16 in HK-2 cells was assessed. **B, G** The CCK-8 assay revealed the proliferation capacity of HK-2 cells after infection with the respective lentivirus. **C, H** The EDU assay demonstrated the proliferation capacity of HK-2 cells after overexpressing or knocking down IFI16 (approximately 200,000 cells). **D, I** The impact of IFI16 overexpression or knockdown on the migration capacity of HK-2 cells. **E, J** The impact of IFI16 overexpression or knockdown on the invasive capacity of HK-2 cells.

*Supplementary Figure 3*.

**A** Top 20 of biological process enrichment. **B** Top 20 of cellular component enrichment. **C** Top 20 of molecular function enrichment. **D** Go enrichment scatterplot.

*Supplementary Figure 4*

**A-D** Typical EDU graphs after overexpression or knockdown of IL6 (approximately 200,000 cells). **E-F** Typical graphs of the scratch assay after overexpression or knockdown of IL6. **G-H** Typical graphs of the transwell assay after overexpression or knockdown of IL6.

*Supplementary Figure 5*.

**A-B** Typical EDU graphs after solely overexpressing IFI16, knocking down IL6, and both overexpressing IFI16 and knocking down IL6 (approximately 200,000 cells). **C-D** Typical graphs of the scratch assay after solely overexpressing IFI16, knocking down IL6, and both overexpressing IFI16 and knocking down IL6. **E** Typical graphs of the transwell assay after solely overexpressing IFI16, knocking down IL6, and both overexpressing IFI16 and knocking down IL6. **F-G** Typical EDU graphs after solely knocking down IFI16, overexpressing IL6, and both knocking down IFI16 and overexpressing IL6 (approximately 200,000 cells). **H-I** Typical graphs of the scratch assay after solely knocking down IFI16, overexpressing IL6, and both knocking down IFI16 and overexpressing IL6. **J** Typical graphs of the transwell assay after solely knocking down IFI16, overexpressing IL6, and both knocking down IFI16 and overexpressing IL6.
