# Supplementary figures and images for: IFI16 promotes the progression of clear cell renal cell carcinoma through the IL6/PI3K/AKT axis
Source: J Transl Med. 2024 Jun 3;22:533. doi: 10.1186/s12967-024-05354-w (PMC11149187; doi:10.1186/s12967-024-05354-w)

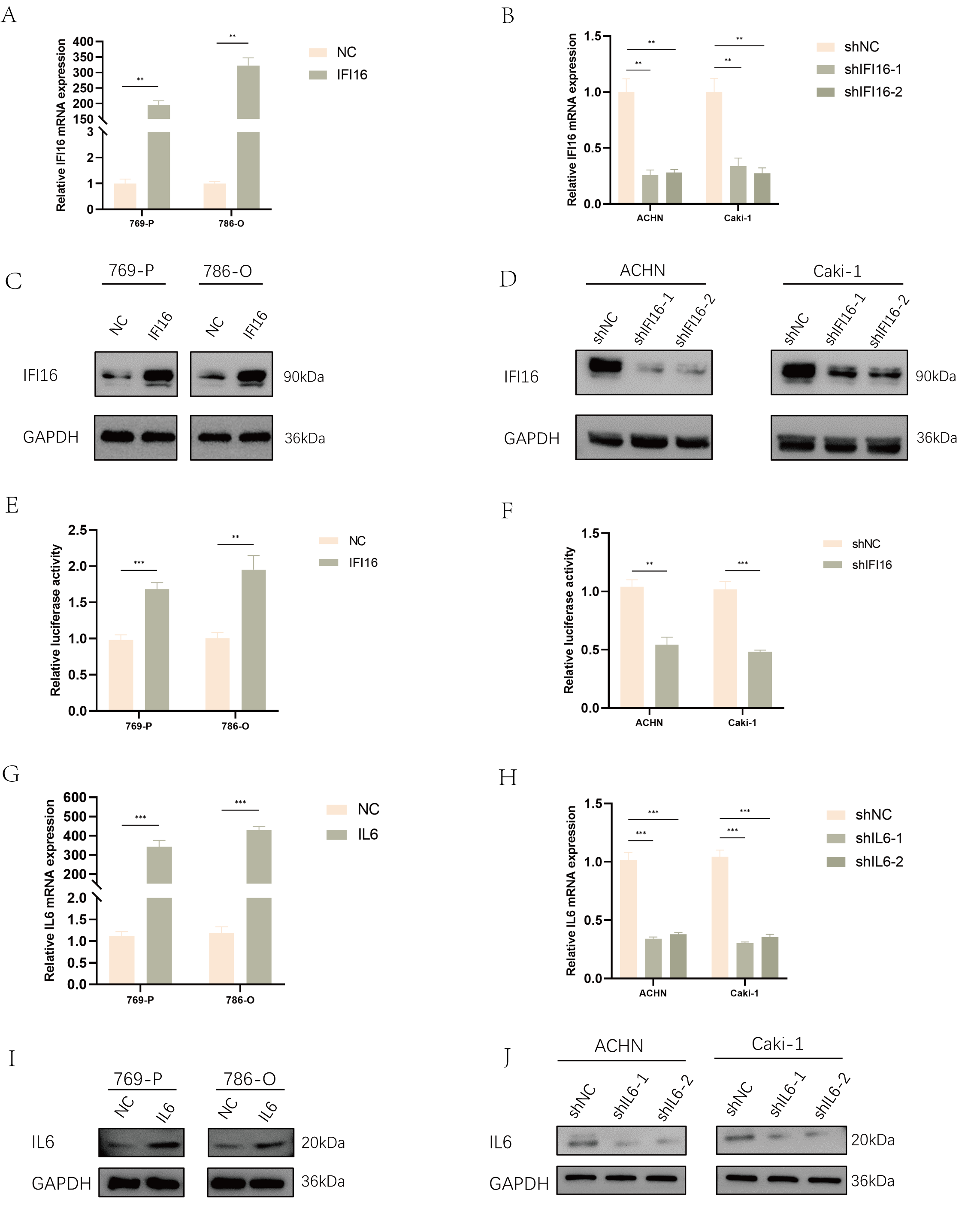

Supplement: Supplementary file 4 — Additional file 4. Figure S1. Validation of stable cell lines and dual-luciferase reporter assay. [file 12967_2024_5354_MOESM4_ESM.tif]

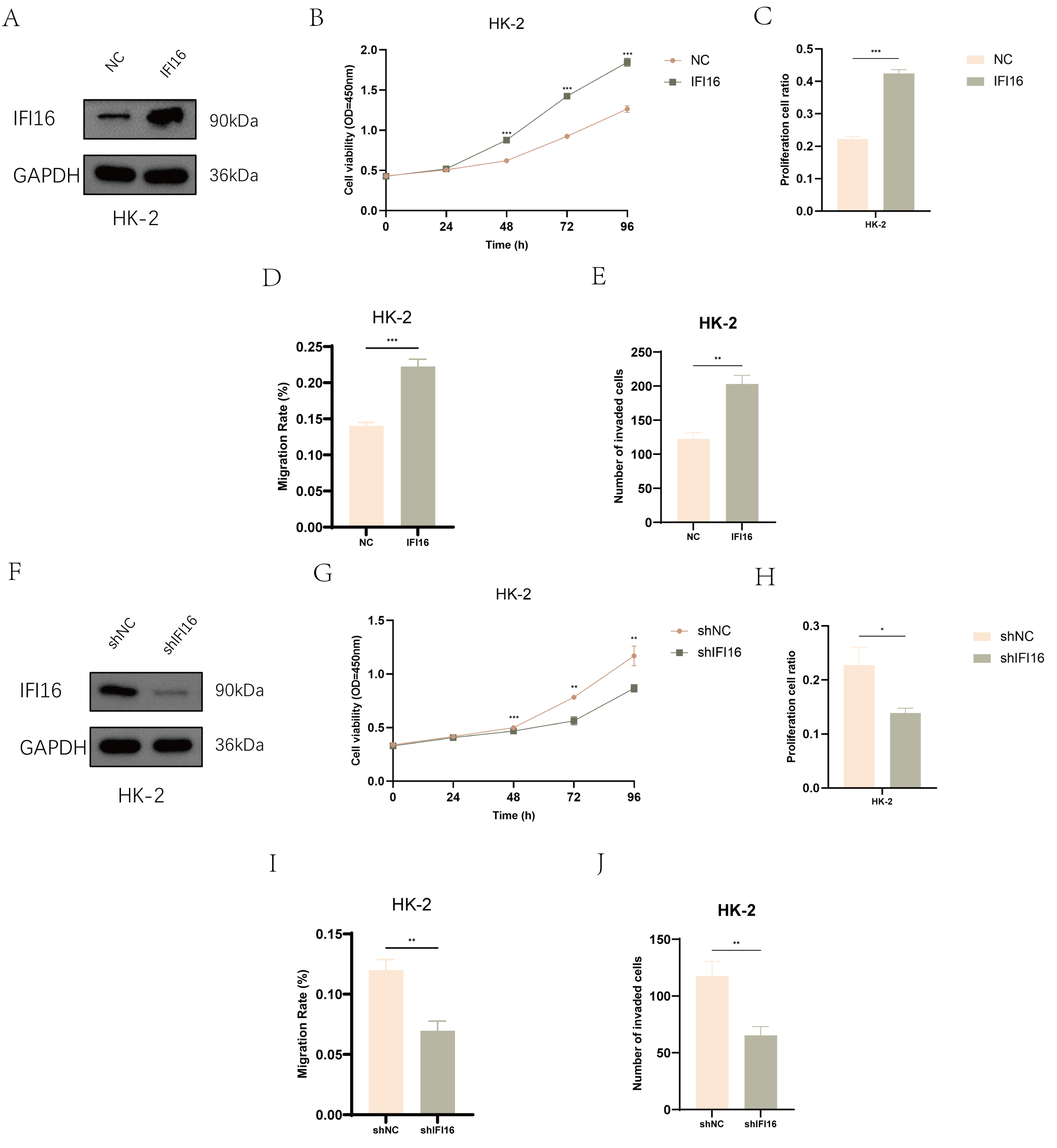

Supplement: Supplementary file 5 — Additional file 5. Figure S2. Validation of stable cell lines and supplementary cell experiments. [file 12967_2024_5354_MOESM5_ESM.tif]

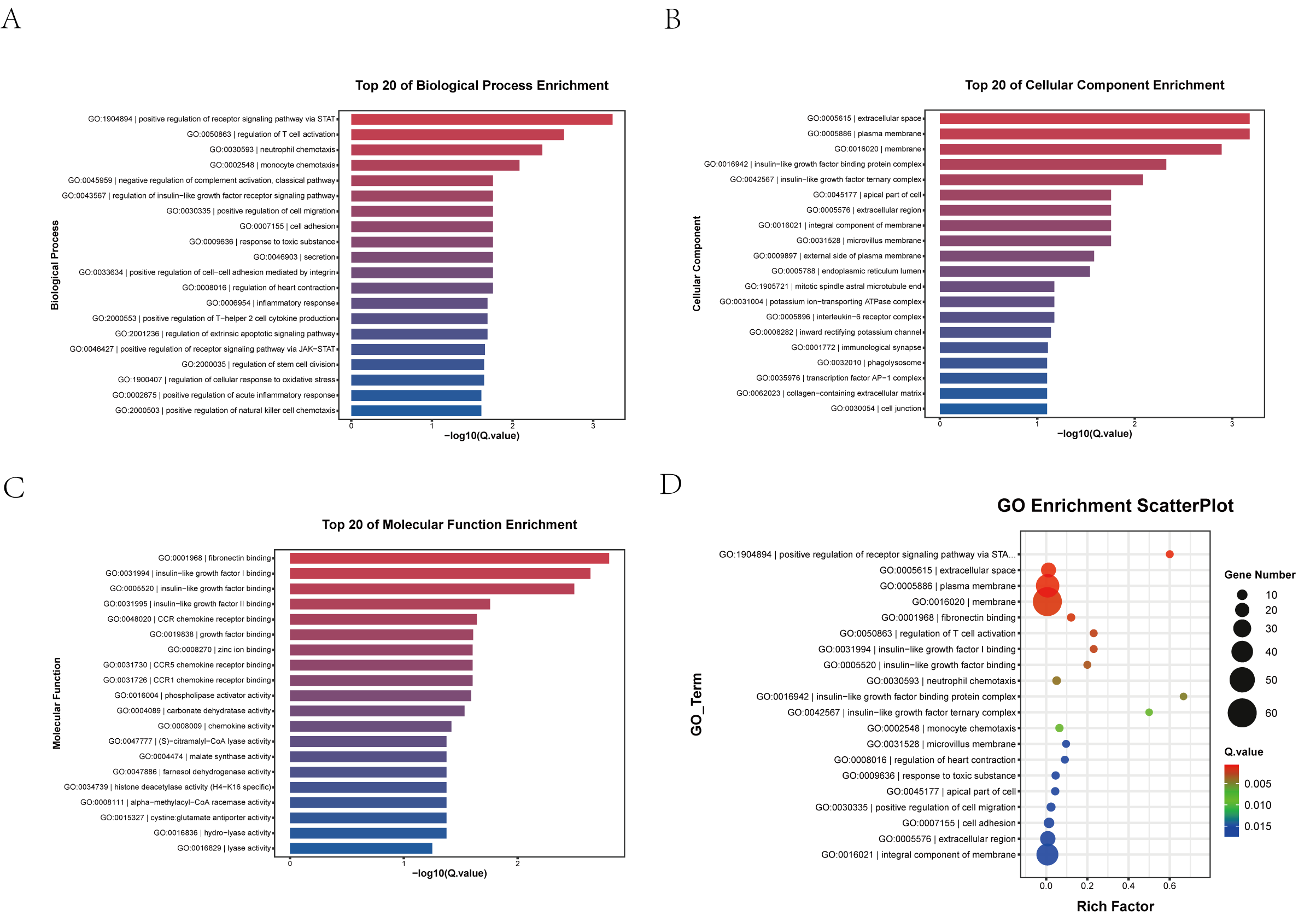

Supplement: Supplementary file 6 — Additional file 6. Figure S3. Enrichment analysis of Gene Ontology. [file 12967_2024_5354_MOESM6_ESM.tif]

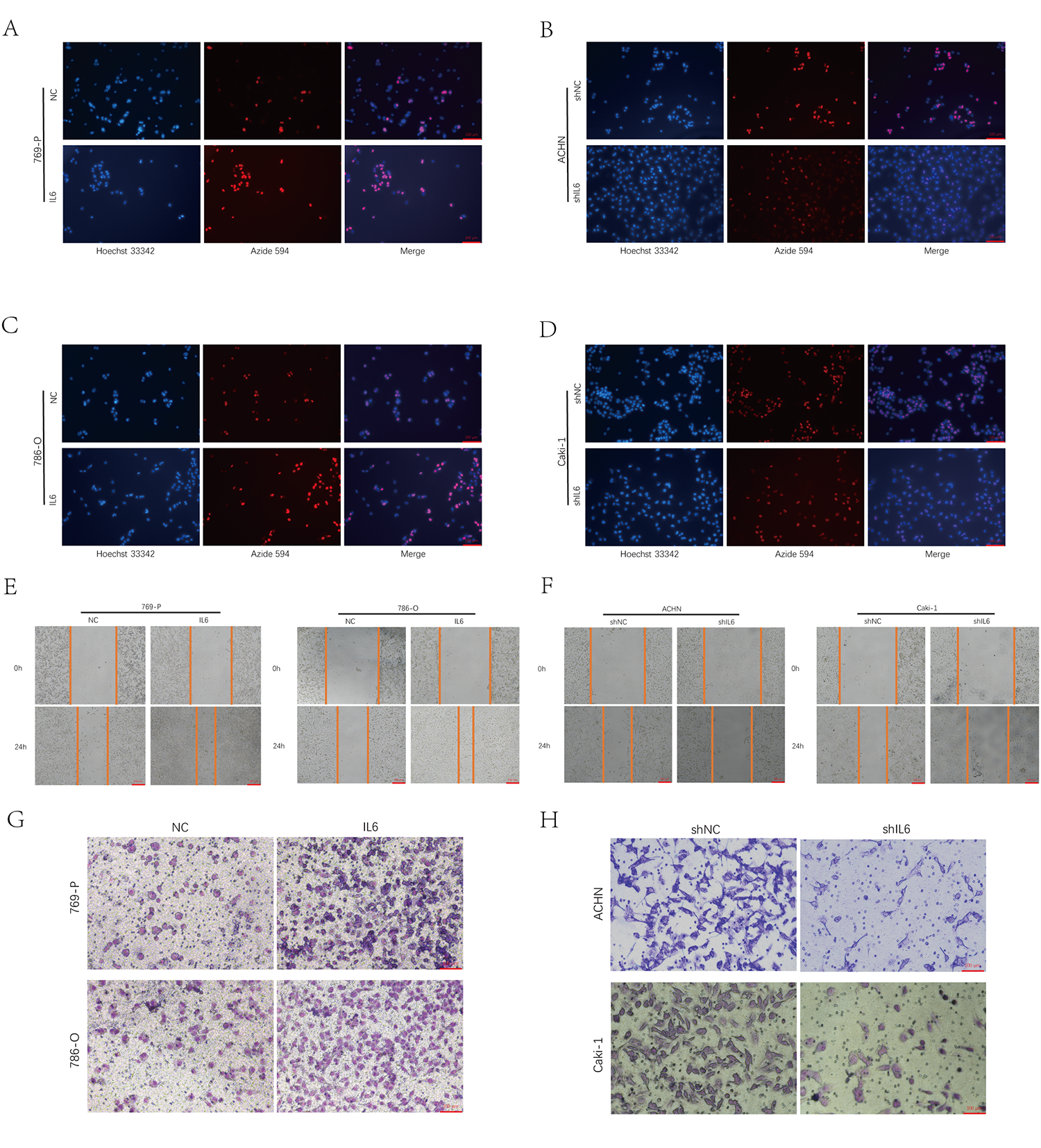

Supplement: Supplementary file 7 — Additional file 7. Figure S4. Typical graphs after overexpression or knockdown of IL6. [file 12967_2024_5354_MOESM7_ESM.tif]

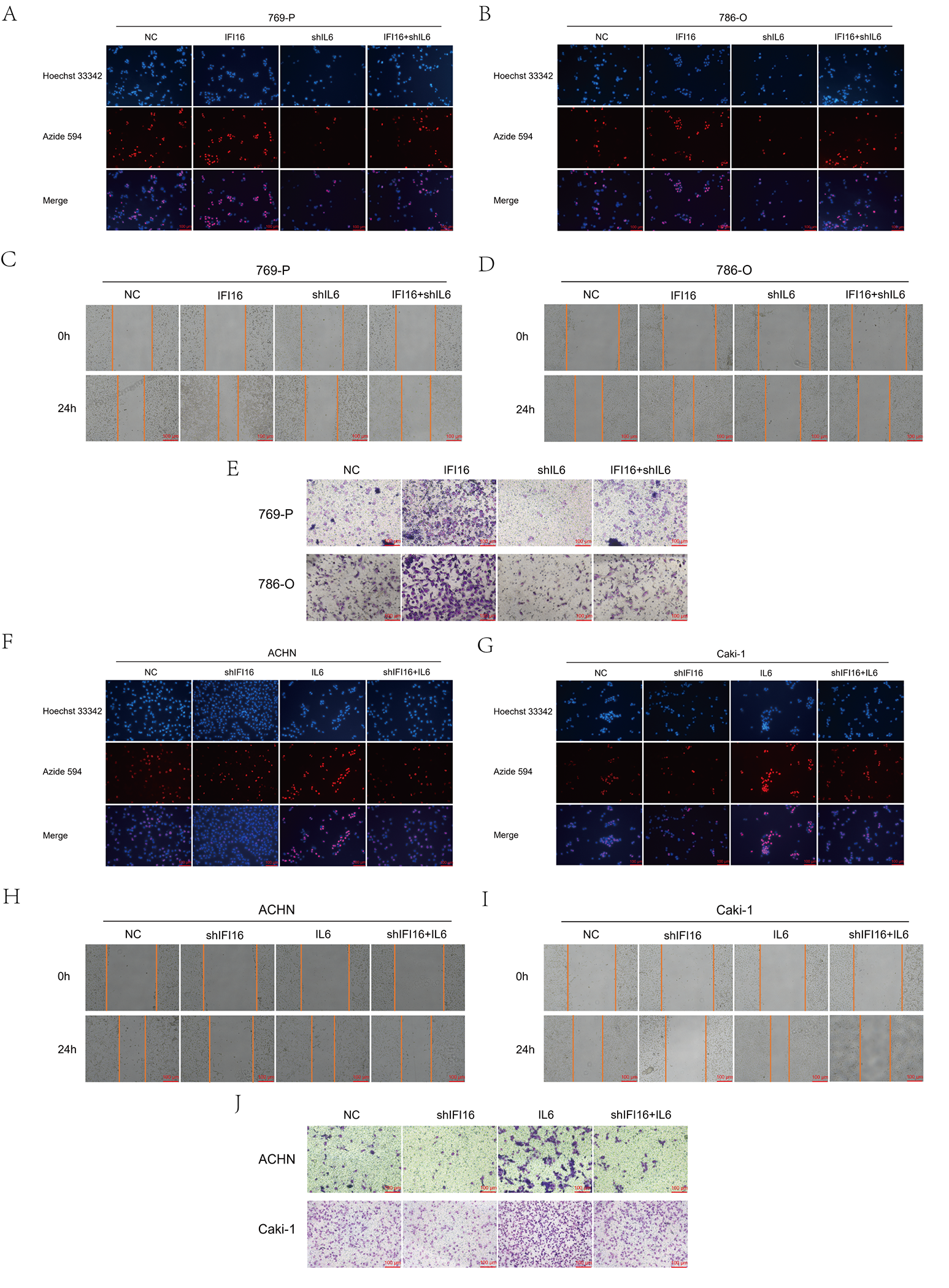

Supplement: Supplementary file 8 — Additional file 8. Figure S5. Typical graphs of the rescue experiments. [file 12967_2024_5354_MOESM8_ESM.tif]
